# Comparing Accuracies of Length-Type Geographic Atrophy Growth Rate Metrics Using Atrophy-Front Growth Modeling

**DOI:** 10.1016/j.xops.2022.100156

**Published:** 2022-04-14

**Authors:** Eric M. Moult, Yingying Shi, Liang Wang, Siyu Chen, Nadia K. Waheed, Giovanni Gregori, Philip J. Rosenfeld, James G. Fujimoto

**Affiliations:** 1 Department of Electrical Engineering and Computer Science, Research Laboratory of Electronics, Massachusetts Institute of Technology, Cambridge, Massachusetts; 2 Health Sciences and Technology, Harvard & Massachusetts Institute of Technology, Cambridge, Massachusetts; 3 Department of Ophthalmology, Bascom Palmer Eye Institute, University of Miami Miller School of Medicine, Miami, Florida; 4 New England Eye Center, Tufts Medical Center, Boston, Massachusetts

**Keywords:** GA, Geographic atrophy, Growth modelling, Growth rate, AREDS, Age-Related Eye Disease Study, GA, geographic atrophy, RPE, retinal pigment epithelium

## Abstract

**Purpose:**

To compare the accuracies of the previously proposed square-root-transformed and perimeter-adjusted metrics for estimating length-type geographic atrophy (GA) growth rates.

**Design:**

Cross-sectional and simulation-based study.

**Participants:**

Thirty-eight eyes with GA from 27 patients.

**Methods:**

We used a previously developed atrophy-front growth model to provide analytical and numerical evaluations of the square-root-transformed and perimeter-adjusted growth rate metrics on simulated and semisimulated GA growth data.

**Main Outcome Measures:**

Comparison of the accuracies of the square-root-transformed and perimeter-adjusted metrics on simulated and semisimulated GA growth data.

**Results:**

Analytical and numerical evaluations showed that the accuracy of the perimeter-adjusted metric is affected minimally by baseline lesion area, focality, and circularity over a wide range of GA growth rates. Average absolute errors of the perimeter-adjusted metric were approximately 20 times lower than those of the square-root-transformed metrics, per evaluation on a semisimulated dataset with growth rate characteristics matching clinically observed data.

**Conclusions:**

Length-type growth rates have an intuitive, biophysical interpretation that is independent of lesion geometry, which supports their use in clinical trials of GA therapeutics. Taken in the context of prior studies, our analyses suggest that length-type GA growth rates should be measured using the perimeter-adjusted metric, rather than square-root-transformed metrics.

Geographic atrophy (GA), also termed complete retinal pigment epithelium (RPE) and outer retinal atrophy,^1^ is the late nonexudative form of age-related macular degeneration. Characterized by contiguous, enlarging regions of atrophied photoreceptors, RPE, and choriocapillaris, GA leads to progressive vision loss.^2^, ^3^, ^4^ Currently, no approved therapeutics exist to stop or slow GA progression, although several recent clinical trials have shown promising results.^5^^,^^6^

Characterizing GA growth is critical both for understanding GA pathophysiologic features and for identifying and evaluating promising GA therapeutics efficiently. Indeed, global GA growth rate is the most commonly used anatomic end point in clinical trials of GA therapeutics.^7^ Although the area-type growth rate (i.e., a growth rate measured in units of area per time, e.g., square millimeters per year) was the first and most common metric of GA growth, it is strongly dependent on baseline lesion size.^8^^,^^9^ This dependency is undesirable because, among other reasons, it complicates growth rate comparisons between differently sized lesions, which, in turn, complicates clinical trial enrollment and comparisons between trials. Yehoshua et al^8^ and Feuer et al^9^ eliminated the dependency of growth rate on baseline lesion size by using the square-root transformation to create a length-type growth metric (i.e., a growth rate measured in units of distance per time, e.g., millimeters per year). Since these initial studies, other length-type growth rate metrics have been proposed, including the effective radius growth metric,^10^ which is a scaled square-root transformation, and the perimeter-adjusted growth metric,^11^ which normalizes the change in lesion area by lesion perimeter. Notably, in Shen et al,^11^ the perimeter-adjusted growth metric was shown to be statistically independent of baseline area, focality, and circularity when evaluated on the Age-Related Eye Disease Study (AREDS) dataset.^11^ Although decoupling growth measurements from aspects of lesion geometry is a reasonable criteria by which to develop and evaluate GA growth metrics, on its own, it provides limited insight into metric accuracy or, more fundamentally, into the biological interpretation of what length-type metrics actually measure. Specifically, although length-type metrics have been motivated by the observation that lesions tend to expand along their margins, length-type growth rates have been interpreted only for simple geometries (e.g., circular lesions) and simple growth patterns (e.g., isotropic growth, that is, growth that is equal in all directions). The absence of an analytical framework within which to interpret and assess length-type growth rates is likely in part a consequence of the complexities of GA geometries and growth patterns, which, although incompletely understood, include:

1. Noncircular margins: Although sometimes starting as circular lesions, GA foci can grow into a variety of complex shapes.
2. Multifocality: GA lesions often comprise multiple foci.
3. Variations in global lesion growth rates: The global (area) rate at which GA lesions enlarge varies among eyes, with some lesions remaining relatively stable and others expanding rapidly.^12^ Although this variability is a topic of current investigation, lesion geometry^13^^,^^14^ and choriocapillaris impairment^15^, ^16^, ^17^, ^18^ have been implicated.
4. Variations in local lesion growth rates: GA lesions do not grow uniformly along their margins.^10^^,^^12^^,^^19^ Currently, it is not known what underlies anisotropic growth.
5. Lesion merging: GA lesions often exhibit merging between different lesion foci (i.e., interfoci merging) and different segments of the same focus (i.e., intrafocus merging).

Incorporating these observations, our group recently developed a mathematical atrophy-front model of GA growth^15^^,^^20^ and demonstrated its usefulness for quantifying local—that is, spatially resolved—GA growth rates. The atrophy-front growth model, so named for its analogy with wildfire propagation, views GA growth as a margin-mediated enlargement process wherein local lesion expansion is determined by an interaction between the lesion’s margin and its chorioretinal milieu (e.g., the choriocapillaris). In the wildfire analogy, the GA lesion margin corresponds to the fire front and the chorioretinal milieu corresponds to the environmental conditions (e.g., fuel sources and winds) that, through interaction with the fire front, help to determine the fire’s spread (of course, this analogy, although useful for gaining a conceptual understanding of the atrophy-front growth model, should not be taken literally). In this article, we use the atrophy-front growth model to provide analytical and simulation-based evaluations of the accuracies of square-root-transformed^8^, ^9^, ^10^ and perimeter-adjusted growth rate^11^ metrics.

## Methods

Below we describe how the atrophy-front model can be used to characterize global length-type GA growth rate metrics rigorously and demonstrate the usefulness of this characterization by exploring some analytical relationships between GA geometry and growth and metric accuracy. Then, we develop simulated and semisimulated datasets of GA lesion growth and describe how the latter can be used to investigate growth metric accuracy under realistic conditions. Throughout, we use the terminology of Table 1 to describe GA geometry and growth. This study was approved by the institutional review board of the University of Miami Miller School of Medicine, was performed in accordance with the tenets of the Declaration of Helsinki, and complied with the Health Insurance Portability and Accountability Act of 1996. All participants provided informed consent.

**Table 1:** Terminology for Describing Geographic Atrophy Geometry and Growth

Global and local growth rates	Global growth rates are single measurements describing how the entirety of a GA lesion expands. Local growth rates are collections of measurements, with each measurement describing how a segment (or point) on the baseline margin expands. In this article, we focus on global growth rates.
Area-type and length-type growth rates	Area-type growth rates describe GA growth in units of area per time, whereas length-type growth rates describe GA growth in units of distance per time. In this article, we primarily focus on global length-type growth rates, which we denote by Λ. We use the notation $\hat{\text{Λ}}$ to denote global length-type growth rate metrics, which we view as estimators of Λ.
Atrophy-front growth model	The atrophy-front growth model describes GA growth as a margin-mediated expansion wherein lesion growth can be described by a growth field, *v*(*x*,*t*), where *x* is the fundus position and *t* is time. This model is stated mathematically in Appendix 1.
Growth field	The growth field, *v*(*x*,*t*), is the local, geometry-independent rate of GA margin enlargement and has units of distance per time. Physiologically, the growth field captures aspects of the chorioretinal milieu that influence GA growth. Larger values of *v* correspond to faster lesion growths.
Time-invariant vs. time-varying growth fields	Time-invariant growth fields do not change in time. In our atrophy-front growth model, this corresponds to *v*(*x*,*t*) = *v*(*x*); that is, the growth field *v* is independent of time. A growth field that is not time invariant is termed *time varying*. For simplicity, in this article, we primarily restrict our attention to time-invariant growth fields.
Isotropic vs. anisotropic growth fields	Isotropic growth fields do not change with spatial position. In the atrophy-front growth model, this corresponds to *v*(*x*,*t*) = *v*(*t*); that is, *v* is independent of position. A growth field that is not isotropic is termed *anisotropic*.

GA = geographic atrophy.

### Geographic Atrophy Growth Rate Measurement as a Problem of Estimating Growth Fields

Given GA lesion margins at a baseline time, *t*_*b*_, and a follow-up time, *t*_*f*_ = *t*_*b*_ + Δ*t*, where Δ*t* is the intervisit time, the global area-type growth rate can be defined unambiguously as (*A*(*t*_*f*_) – *A*(*t*_*b*_)) / Δ*t*, where *A*(*t*) denotes the GA area at time *t*. In contrast, no single notion of length-type growth rate exists. For example, depending on the application, it might be sensible to construct a length-type growth rate using closest extrinsic (e.g., Euclidean, that is, straight-line) distances,^21^ perimeter normalizations,^11^ or specialized functions.^22^ Consequently, before computing the accuracies of different length-type growth rate metrics, first it is necessary to make precise what notion of length-type growth rate will be used. Because different notions of length-type growth rates yield different measurements and carry different biological interpretations, it is desirable to use a formulation that is consistent with the known or hypothesized processes underpinning GA growth. Toward this end, in this study, we took as our starting point the atrophy-front growth model, which our group previously proposed as a physiologically plausible description of GA growth.^15^ The atrophy-front model of GA growth is a mathematical description of the margin-mediated growth hypothesis, whereby existing GA regions are hypothesized to expand along their borders of atrophy. The margin-mediated growth mechanism is supported by, or at least consistent with, the clinical observation that regions of new atrophy accumulate predominantly, although not exclusively, along the margins of existing foci. Moreover, the margin-mediated growth mechanism agrees with the finding that area-type GA growth rate is correlated strongly with lesion perimeter,^11^ that is, more margin corresponds to more (area) growth. Although the mechanisms of margin-mediated growth are unknown, death signaling from RPE cells undergoing apoptosis or necroptosis^23^ and the necessity of RPE–RPE cell signaling^24^ have been suggested.

Importantly, it can be shown (Appendix 1) that existing length-type growth rate metrics—namely, the square-root-transformed^8^, ^9^, ^10^ and perimeter-adjusted growth rate^11^ metrics—can be derived from the atrophy-front model of GA growth. Specifically, these existing metrics correspond to estimates of the length-type growth rate, Λ, defined as a position-time average of a 2-dimensional (i.e., en face) time-varying growth field, *v*(*x*,*t*) (Appendix 1). The growth field can be understood as a mathematical abstraction that encodes the state of the chorioretinal milieu, that is, the intracellular and extracellular environment of the choroid and retina, which includes RPE and photoreceptor integrity, as well as the choriocapillaris blood flow. Chorioretinal conditions leading to faster growth correspond to higher values of *v*, and vice versa. In the wildfire analogy of GA growth, the growth field corresponds to the environmental conditions that, through interaction with the fire front, help to determine the fire’s spread.

A benefit of viewing growth rate metrics through the lens of the atrophy-front growth model is the ability to derive relationships between GA geometry, GA growth patterns, and metric accuracy. For example, consider the effective radius metric, ${\hat{\text{Λ}}}_{ER}$, defined as^10^:

$${\hat{\text{Λ}}}_{ER}\equiv \frac{\sqrt{A(t_{f})\;}-\;\sqrt{A(t_{b})}}{\sqrt{\pi }\text{Δ}t},\;\left(1\right)$$

where *A*(*t*) is the lesion area at time *t*. Note that the effective radius metric is simply a 1 / $\sqrt{\pi }$ scaled version of the square-root-of-area metric.^8^^,^^9^ Although in this article we work with the effective radius metric, corresponding results for the square-root-of-area metric are obtained through a $\sqrt{\pi }$ scaling. As suggested by its name, ${\hat{\text{Λ}}}_{ER}$ assumes that the baseline and follow-up lesions are concentric circles and measures the difference of their radii; if this strict geometrical condition is satisfied, then it is easy to show that ${\hat{\text{Λ}}}_{ER}$ = *v* = Λ for isotropic, time-invariant growth fields; that is, under such conditions, ${\hat{\text{Λ}}}_{ER}$ is a perfect estimator of length-type growth rate. For more complex lesion geometries, ${\hat{\text{Λ}}}_{ER}$ becomes a worse estimator of Λ. For example, for lesion growths that are small relative to the baseline margin perimeter *P*(*t*_*b*_) and baseline area *A*(*t*_*b*_), it can be shown (Appendix 2) that Λ and ${\hat{\text{Λ}}}_{ER}$ are related by:

$${\hat{\text{Λ}}}_{ER}\approx \frac{\text{Λ}}{\sqrt{circ\left(G\left(t_{b}\right)\right)}},\;\left(2\right)$$

where *G*(*t*) is the GA lesion geometry at time *t* and circ(·) is the circularity operator, defined as: circ(*G*(*t*)) ≡ 4π*A*(*t*) / *P*^2^(*t*), which takes values between 0 and 1, inclusive. Note that this is the same definition of circularity as used by Domalpally et al^14^ in their study of GA growth rates. The accuracy of ${\hat{\text{Λ}}}_{ER}$ also degrades when measuring multifocal lesions. For example, for *n* equal-radii circular foci undergoing isotropic growth:

$${\hat{\text{Λ}}}_{ER}=\sqrt{n}\;\text{Λ}\;\text{, }\left(3\right)$$

Observing that GA lesions tend to enlarge along their margins, researchers proposed the perimeter-adjusted growth rate metric, ${\hat{\text{Λ}}}_{PA}$, as an extension of the effective radius growth rate to more general, noncircular geometries^11^:

$${\hat{\text{Λ}}}_{PA}\equiv {\left(\frac{P\left(t_{f}\right)\;+\;P\left(t_{b}\right)}{2}\right)}^{-1}\frac{A\left(t_{f}\right)\;-\;A\left(t_{b}\right)}{\text{Δ}t}.\;\left(4\right)$$

Conveniently, this metric has been well studied in the computer-aided geometric design community in the context of offset curves. It can be shown^25^ that for isotropic, time-invariant growth fields, ${\hat{\text{Λ}}}_{PA}\;$ = Λ for lesions having concave curvatures whose absolute values are small relative to the lesion growth (an illustration of concave and convex margin segments, as well as the precise definition of small, is provided in Appendix 3 and Figure S1). Briefly and informally, a concave margin segment corresponds to an inward bulge in the lesion, and a convex margin segment corresponds to an outward bulge in the lesion). Intuitively, this condition requires that no intrafocus merging occurs. Of note, ${\hat{\text{Λ}}}_{PA}\;$ = Λ for any convex lesion undergoing isotropic, time-invariant growth (a lesion is convex if the entirety of its margin is convex, that is, the lesion has no inward bulges; for example, ellipse-shaped lesions are convex).

Although the above results demonstrate the usefulness of the atrophy-front growth model for interpreting length-type growth rate metrics, they pertain to simplistic geometries and growth patterns. Analysis of arbitrary geometries and growth patterns, for which analytical results are unwieldy or intractable, can be approached numerically, which is the focus of the subsequent section.

### Generating Datasets for Numerical Evaluation of Metric Accuracy

Compared with clinically observed GA data, for which the complete growth dynamics are not well understood, simulated GA data have the advantage that the true growth parameters can be precisely specified. Importantly, an observed data set comprising images of a GA lesion acquired at a sequence of follow-up visits does not provide direct information related to the underlying growth field; different growth fields can result in the same observed growth data. This ambiguity is particularly acute for common clinical datasets, for which the intervisit times are relatively lengthy (e.g., 6 months or longer). Because of these challenges, we outline below our growth simulation approach, and then we construct a set of highly simplified lesion geometries and growth fields that highlight inherent limitations of the square-root-transformed and perimeter-adjusted metrics. Finally, we present a semisimulated approach to generating realistic GA growth configurations using real lesion geometries and simulated random growth fields.

#### Simulating Lesion Growths and Computing Metric Accuracies

**Figure 1:**
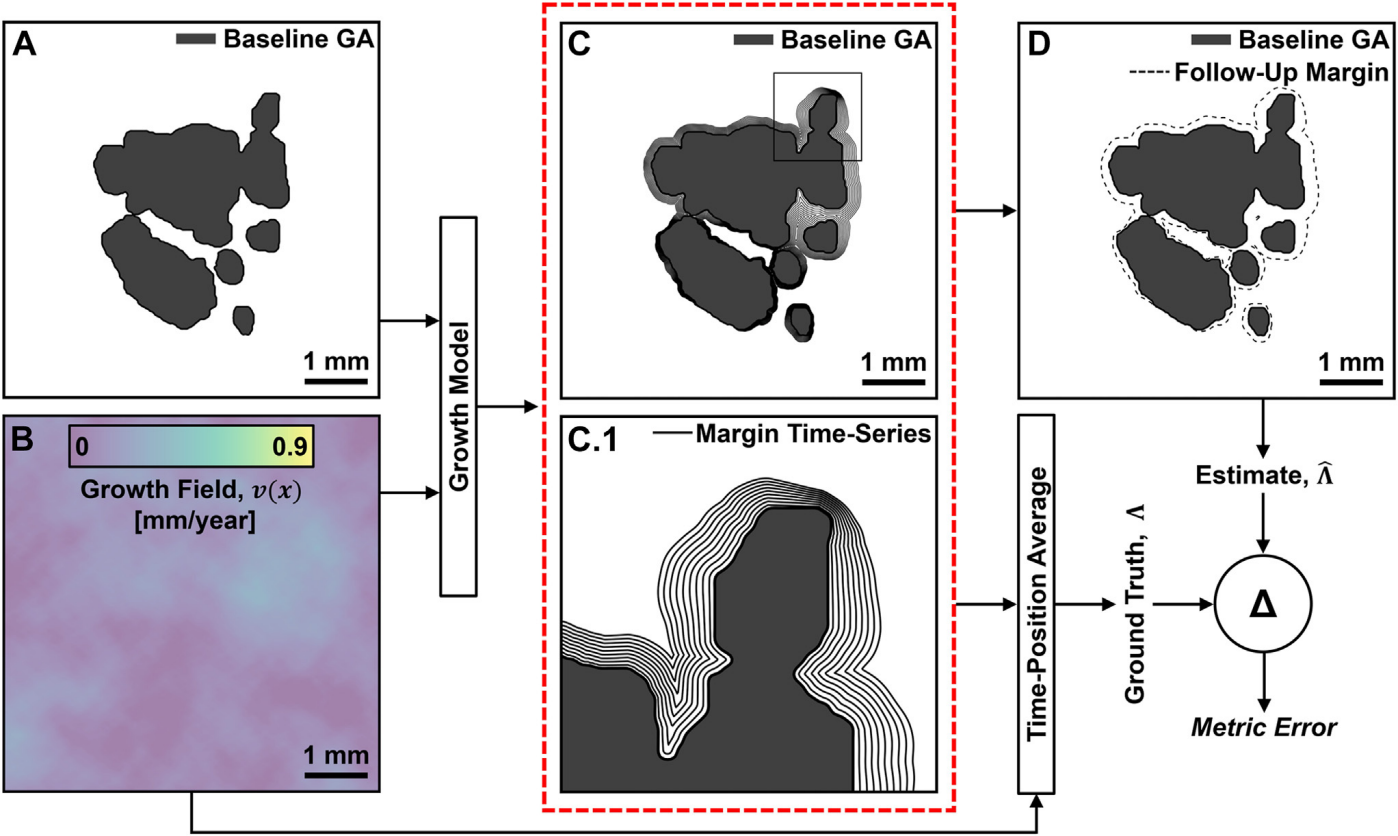
Illustration of the method for assessing growth rate metric accuracy. The geographic atrophy (GA) growth model (equation SI-2, Appendix 1) uses (**A**) baseline lesion data, either simulated or observed, and (**B**) a simulated growth field to produce (**C**) a sequence of GA margins. This sequence of GA margins, a portion of which is enlarged in (**C.1**), comprises simulated margins at different time points equally spaced between the baseline and follow-up visits. From this sequence of GA margins, (**D**) the baseline and follow-up margins can be extracted, mimicking clinically available GA growth data. Using the sequence of GA margins and the growth field data, the ground truth global length-type growth rate, Λ, can be computed (equation SI-3, Appendix 1). A growth metric $\hat{\text{Λ}}$ uses the baseline and follow-up GA margins (**D**) to produce a growth rate measurement that can then be compared against the ground truth Λ.

The process of generating simulated GA growth data and computing the associated metric accuracies is shown schematically in Figure 1. Briefly, baseline lesion contours, either simulated or observed, are propagated outward (see equation SI-2 of Appendix 1) according to a simulated growth field during the intervisit time Δ*t*. This propagation yields a sequence of GA margins, the last of which corresponds to the follow-up GA margin. Using the sequence of GA growth margins in conjunction with the simulated growth field, the ground truth global length-type growth rate Λ can be computed (via equation SI-3 of Appendix 1). Furthermore, mimicking the clinical situation, growth rate estimates $\hat{\text{Λ}}$ can be computed using only the baseline and follow-up GA margins, via equation 1 (${\hat{\text{Λ}}}_{ER}$) or equation 4 (${\hat{\text{Λ}}}_{PA}$). Metric accuracy then is assessed by comparing the growth rate estimates $\hat{\text{Λ}}$ with the ground truth growth rates Λ. Details of the numerical implementations of these steps are provided in Appendix 4. Note that, for simplicity, in all of our analyses we restrict our attention to time-invariant growth fields.

#### Simulations with Simplified Lesion Geometries and Growth Fields

**Figure 2:**
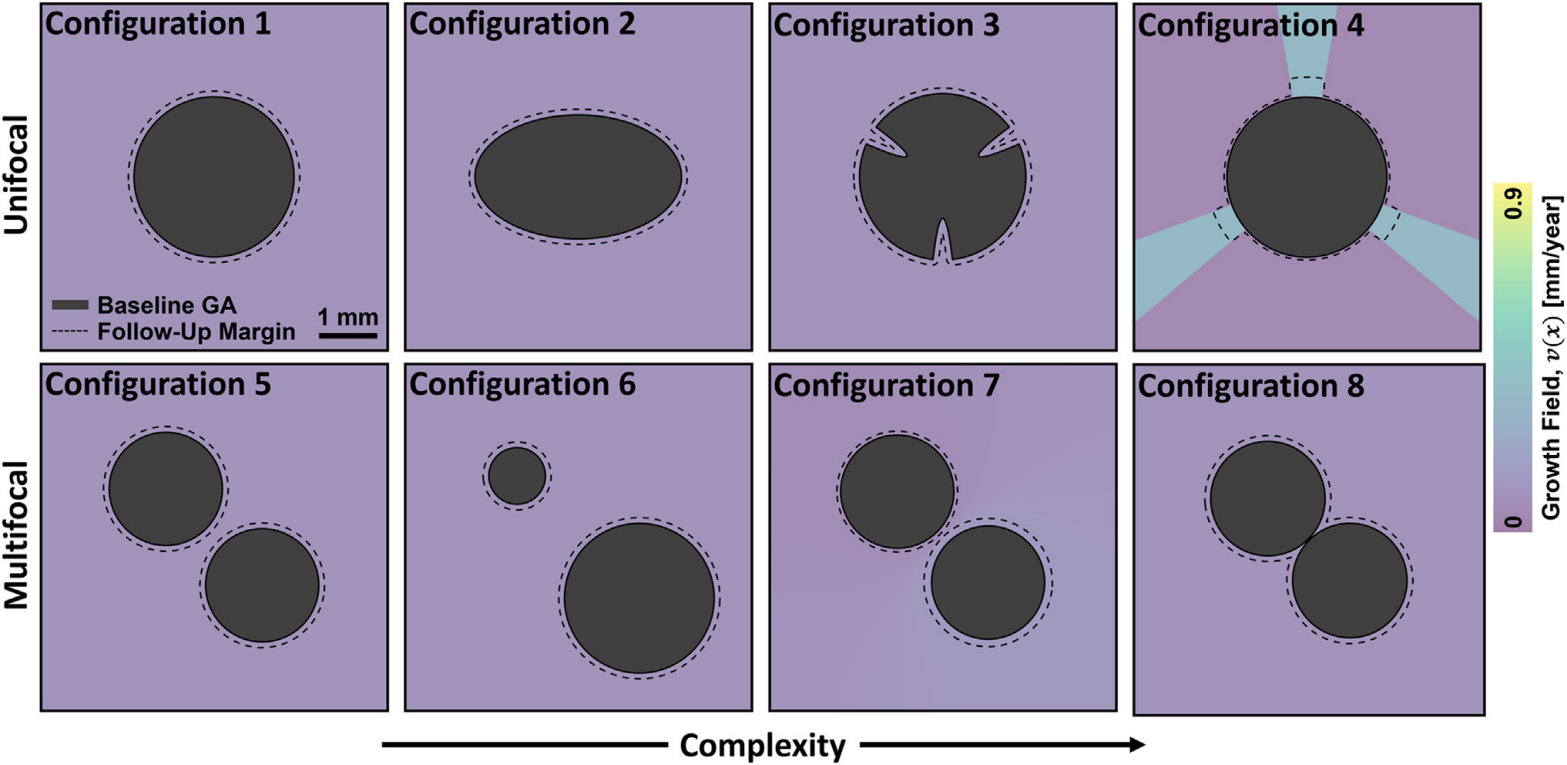
Simple simulated lesion geometries and growth fields constructed to highlight key characteristics of global length-type growth rate metrics. Configurations 1 through 8 correspond to different lesion geometries and growth fields. All lesions were constructed to have baseline areas of 6 mm^2^ and all growth fields were constructed to have a length-type growth rate of Λ = 0.1 mm/year, as measured over a 1-year follow-up time. Colors correspond to growth field values (see color bar). Note that all configurations except for configurations 4 and 7 have isotropic growth fields. Mathematical details of these configurations are provided in Appendix 5.

Figure 2 illustrates a hierarchy of simplified, simulated lesion geometries and growths whose rows are arranged according to focality and whose columns are arranged according to a subjective complexity of lesion geometry and growth pattern. All lesions of Figure 2 were constructed to correspond to Δ*t* = 1 year follow-up intervals to have equal baseline areas of *A*(*t*_*b*_) = 6 mm^2^ and to have equal length-type growth rates of Λ = 0.1 mm/year, the latter of which approximately corresponds to the reported mean of the 1-year perimeter adjusted growth rates of the AREDS dataset.^11^ Mathematical specifications of the lesion geometries and growth fields are given in Appendix 5. Using the terminology of Table 1, configurations 1 through 3 represent a progression of geometric complexity, moving from a circle (configuration 1) to an ellipse (configuration 2) to a notched shape (configuration 3). Relevant to growth rate estimation, configurations 1 and 2 both are convex shapes, and therefore isotropic growth never results in intrafocus merging (Appendix 3); however, configuration 2 is noncircular (i.e., has a circularity index of < 1), and therefore results in errors when assessed using square-root-transformed metrics (equation 2). Configuration 3 has a nonconvex geometry, and therefore, lesion growth can lead to intrafocus merging, which has the effect of censoring GA growth, making growth estimation ill-posed for any metric (Appendix 3). Compared with configurations 1 through 3, configuration 4 corresponds to an anisotropic growth field, which causes the baseline circular lesion to grow into a more complex, nonconvex geometry. In the second row, although the baseline lesion shapes are all circular, bifocality results in estimation errors when using square-root-transformed metrics (equation 3). Configurations 5 and 6 correspond to different distributions of lesion area between the foci. Configuration 7 corresponds to an anisotropic growth field, constructed so that the lesion growth rate of each lesion focus is constant, but with the rate of the top left lesion being half that of the bottom right lesion. Finally, unlike configurations 5 through 7, configuration 8 involves merging of the 2 lesion foci (i.e., interfoci merging). Evaluations of metric accuracy were performed by scaling the growth fields (but not the baseline lesion geometries) so as to vary Λ through a range of 0.05 to 0.7 mm/year (Appendix 5).

#### Simulations with Observed Lesion Geometries and Random Growth Fields

To assess the accuracy of ${\hat{\text{Λ}}}_{ER}$ and ${\hat{\text{Λ}}}_{PA}$ under more realistic conditions, we generated semisimulated GA growth data using clinically observed lesion geometries and simulated random growth fields. This hybrid approach is attractive because it captures the full complexities of (baseline) GA geometries and, through judicious construction of the simulated growth fields, generates GA growth patterns with features that mimic many characteristics of GA growth in vivo. Although details are provided in Appendix 6–Appendix 8, briefly, our approach to generating semisimulated GA growth data consists of: (1) extracting a set of baseline GA lesions from clinical OCT imaging data, (2) generating random growth fields having characteristics that match the measurements of in vivo observations, and (3) propagating outward the baseline lesion geometries using the random growth fields, as above, to obtain a follow-up lesion margin.

Baseline lesion geometries were extracted from a 6 × 6-mm field-of-view OCT imaging dataset of 38 GA eyes (27 patients). These imaging data were used in a prior study of GA growth rate by our group,^19^ and we refer readers to this prior publication for detailed information regarding enrollment criteria, patient demographics, and acquisition; details of lesion processing for the simulations of this study are provided in Appendix 7.

Random growth fields were constructed to have the following features: (1) variations in global growth rates, (2) variations in local growth rates, and (3) global growth rate statistics approximating those of clinically reported data. In particular, regarding the third feature, we approximately matched the mean (μ_Λ_) and standard deviation (σ_Λ_) of the simulated global growth rates to those of the 1-year perimeter-adjusted growth rates of the AREDS dataset,^11^ namely, μ_Λ_ ≈ σ_Λ_ ≈ 0.1 mm/year. As described in Appendix 6, to augment our dataset, for each of the 38 baseline lesions, 100 random fields were generated, resulting in a total of 3800 baseline and follow-up margin pairs with which to evaluate metric accuracy for a given follow-up interval (Δ*t*). Finally, we assessed metric accuracy at follow-up time intervals of Δ*t* = 1, 3, and 5 years.

## Results

**Figure 3:**
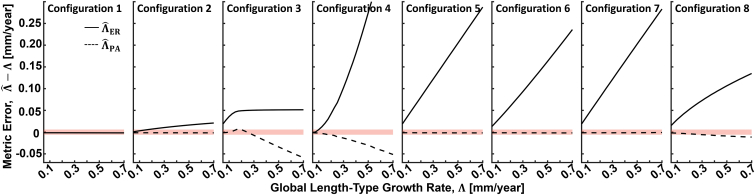
Accuracy of global length-type growth rate metrics as evaluated on the simple lesion geometries and growth fields of Figure 2. Note that although Figure 2 shows growth fields for a fixed growth rate of Λ = 0.1 mm/year, as described in the text, the growth fields were scaled to assess metric accuracy as a function of the growth rate Λ. The thick red line corresponds to a range of ± 6 μm, corresponding to the half-width of the 12-μm pixel size used for these simulations (Appendix 5). The solid and dashed lines correspond to the effective radius ${\hat{\text{Λ}}}_{ER}$ and perimeter-adjusted ${\hat{\text{Λ}}}_{PA}$ growth rates, respectively. For all plots, the intervisit time, Δ*t*, was 1 year.

Figure 3 shows the effective radius ${\hat{\text{Λ}}}_{ER}$ and the perimeter-adjusted ${\hat{\text{Λ}}}_{PA}$ metrics plotted against the ground truth growth rates Λ for each of the configurations of Figure 2. We can see that, as was analytically derived in the Methods, the perimeter adjusted growth rate ${\hat{\text{Λ}}}_{PA}$ is a perfect estimator of Λ for isotropic, nonmerging growth patterns (configurations 1, 2, 5, 6, and 7). We also see that, even for configurations violating these requirements (configurations 3, 4, and 8), ${\hat{\text{Λ}}}_{PA}$ performs at or near the pixel resolution of the simulation grid (± 6 μm; Appendix 5) for most clinically observed growth rates. In comparison, the effective radius metric ${\hat{\text{Λ}}}_{ER}$ performs relatively poorly for all but the simplest configuration (configuration 1), for which it was designed. Specifically, the effective radius metric is affected by circularity (equation 2), number of lesion foci (equation 3), and lesion merging.

**Figure 4:**
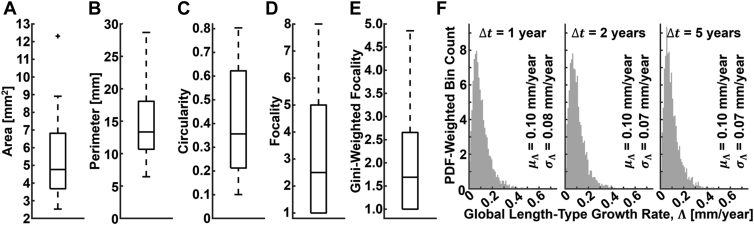
Lesion and growth field characteristics for the semisimulated metric analysis. **A**–**E**, Characteristics of the baseline lesion data for the 38 eyes with geographic atrophy. Gini-weighted focality, which adjusts for situations in which the foci have unequal perimeters, is described in Appendix 9. **F**, Probability distribution function (PDF)-weighted histograms of the ground truth global length-type growth rate Λ for the Δ*t* = 1-, 3-, and 5-year simulations. Distribution means (μ_Λ_) and standard deviations (σ_Λ_) also are provided. As expected, the distributions, means, and standard deviations are very similar for the Δ*t* = 1-, 3-, and 5-year simulations. For all boxplots, whiskers extend beyond the box edges to a maximum of ×1.5 the interquartile range; measurements beyond this range are indicated by crosses. Histogram bins were chosen using the Freedman-Diaconis rule.

**Figure 5:**
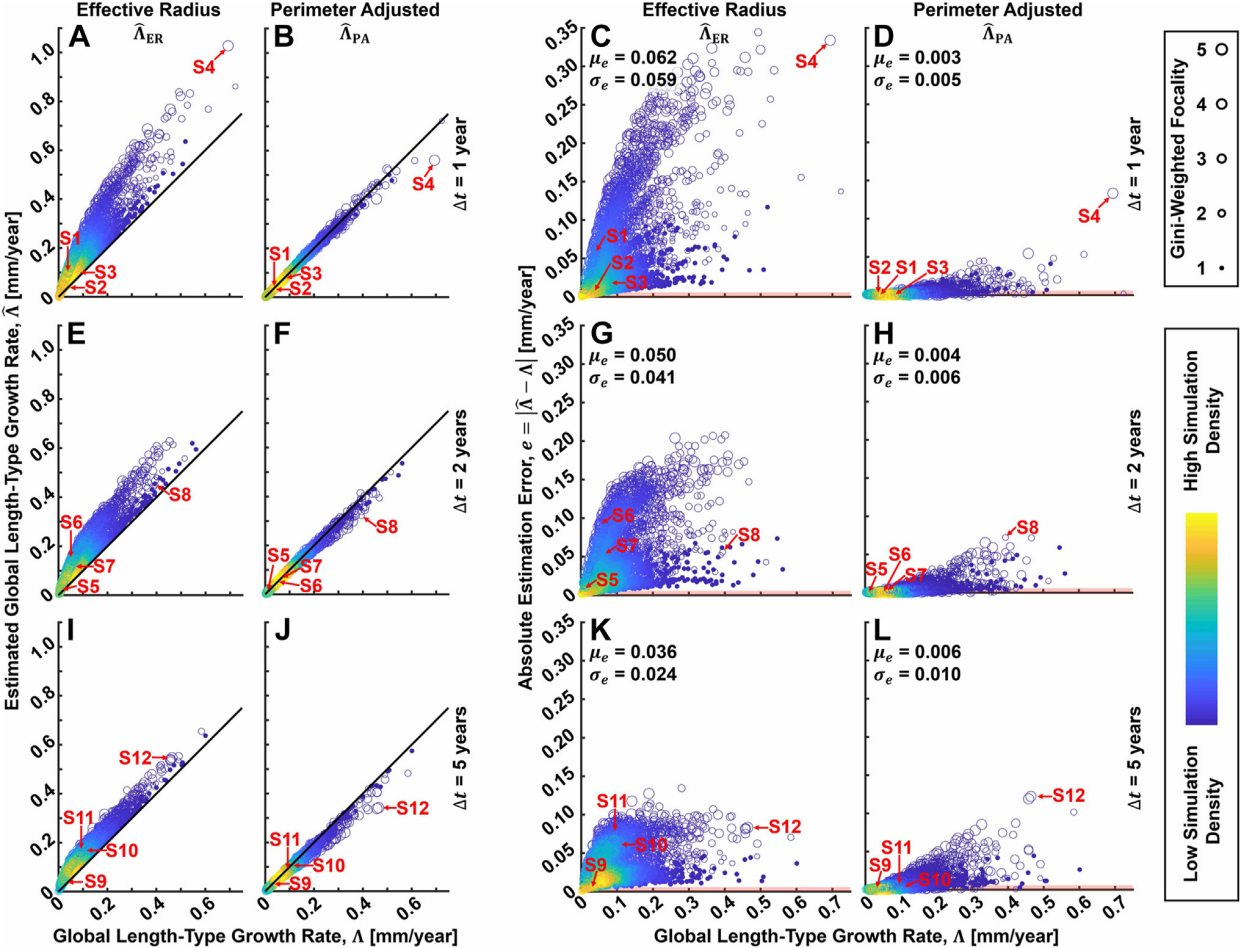
Summary of effective radius and perimeter-adjusted metric accuracies as evaluated on the semisimulated geographic atrophy (GA) dataset. **A**, **B**, Density scatterplots of the estimated growth rates versus the ground truth growth rates, evaluated on 1-year intervisit times. For all panels, each marker represents a single growth simulation (i.e., 3800 markers per panel). Colors correspond to the relative density of measurement points, and marker sizes correspond to the Gini-weighted focality (see legend, far right; Gini-weighted focality is described in Appendix 9). **C**, **D**, Density scatterplots of the absolute estimation error, *e*, evaluated on 1-year intervisit times. The mean (μ_*e*_) and standard deviation (σ_*e*_) of the absolute estimation errors are listed. **E**–**H**, **I**–**L**, Analogous panels, but corresponding to 2-year (**E**–**H**) and 5-year (**I**–**L**) intervisit times. The labels S1 through S12 correspond to representative cases that are explored in Figure 6. In particular, for each intervisit time, simulations generating absolute perimeter adjusted errors at the twenty-fifth (S1, S5, S9), fiftieth (S2, S6, S10), seventy-fifth (S3, S7, S11), and one-hundredth (S4, S8, S12) percentiles were selected.

**Figure 6:**
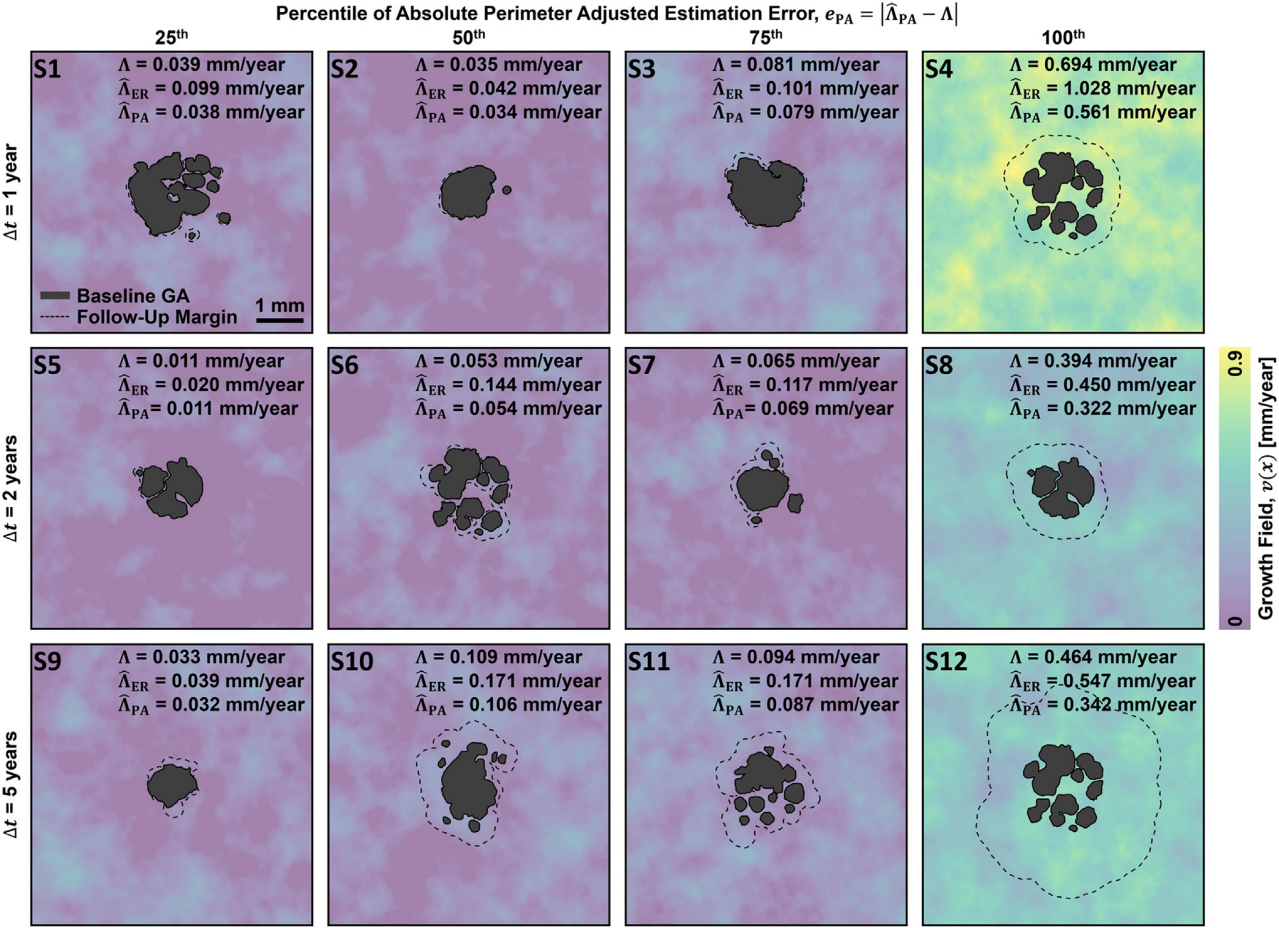
Representative semisimulated geographic atrophy (GA) growth data, with panels S1 through S12 corresponding to the labels of Figure 5. Each column corresponds to simulations with absolute perimeter adjusted errors, *e*_*PA*_, of the specified percentile, and each row corresponds to simulations of the specified intervisit time.

**Figure 7:**
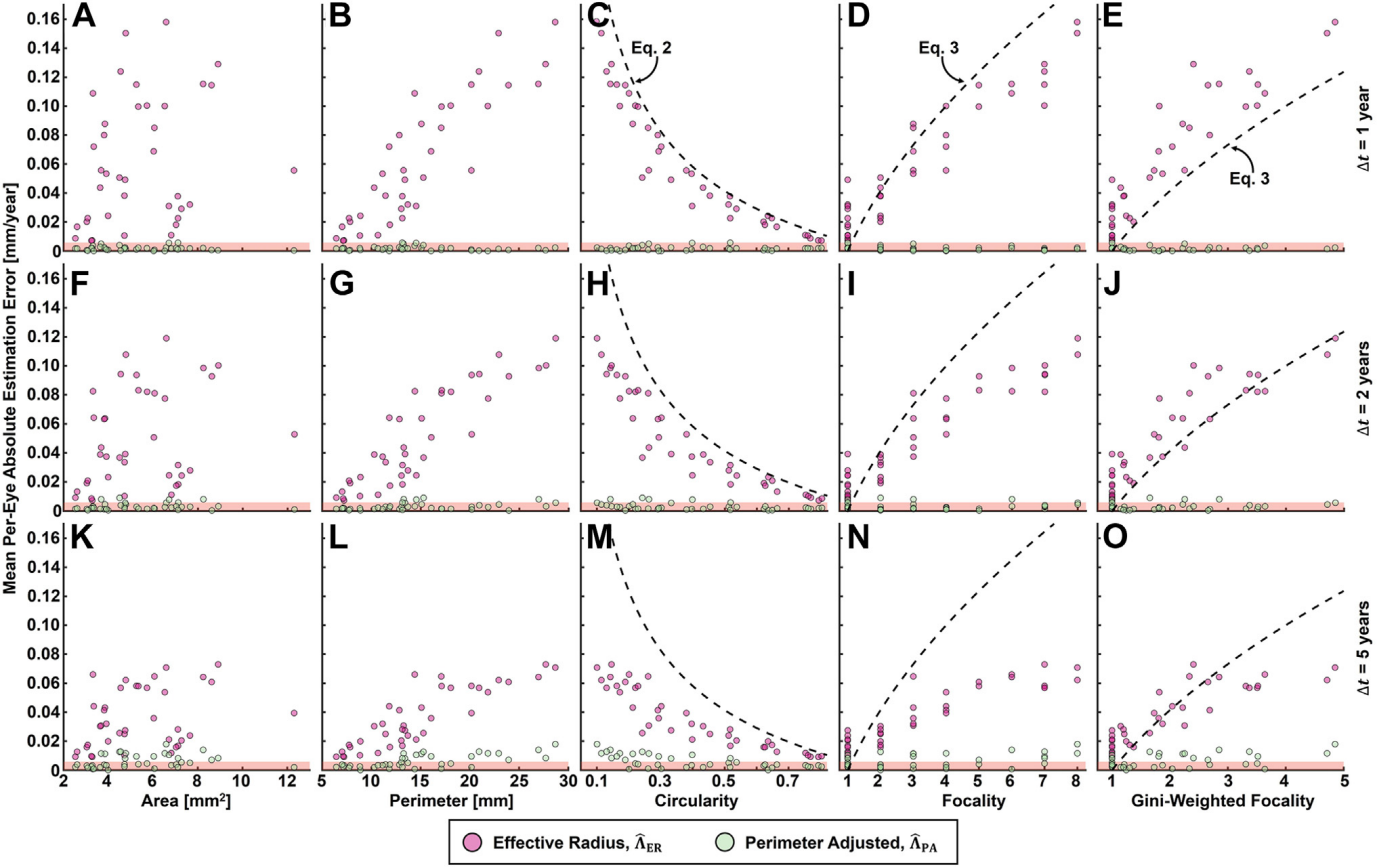
Scatterplots showing the mean per-eye absolute estimation errors as a function of baseline lesion descriptors for the semisimulated analyses. For all panels, each marker corresponds to the mean absolute error $\left|\hat{\text{Λ}}-\text{ Λ}\right|$ for a single baseline lesion, where the average is computed over the 100 simulated random fields applied to that lesion (i.e., 38 green and 38 pink markers per panel, corresponding to the effective radius and perimeter-adjusted measurements, respectively). In columns 3 through 6, the dashed lines correspond to the errors predicted by equations 2 and 3. Gini-weighted focality is described in Appendix 9.

Figure 4 summarizes the baseline characteristics of the 38 GA lesions used for the semisimulated growth data. Note that the Gini-weighted focality (Fig 4E) is a continuous-valued measure of focality that adjusts for different foci having different perimeters. In particular, the Gini-weighted focality is equal to the number of foci if all the foci have equal perimeters, but is less than the number of foci if the foci have unequal perimeters; moreover, the more unequal the perimeters, the lower the Gini-weighted focality. The Gini-weighted focality is described further in Appendix 9. The distributions of the simulated ground truth growth rates for the 3800 growth simulations are provided for the 3 tested follow-up intervals (Δ*t* = 1, 2, and 5 years). The statistics of these distributions match fairly well those of the perimeter-adjusted growth rates reported for the AREDS dataset, although the standard deviations are slightly smaller (approximately 0.07 mm/year for our simulations and approximately 0.10 for the AREDS dataset), presumably a consequence of the averaging involved in computing Λ (equation SI-3, Appendix 1). Figure 5 shows the accuracies of ${\hat{\text{Λ}}}_{ER}$ and ${\hat{\text{Λ}}}_{PA}$ evaluated on this semisimulated growth data. The perimeter-adjusted metric shows a marked improvement in accuracy compared with the effective radius metric (approximately 20 times improvement in average accuracy for the 1-year follow-up data). Notably, for nearly all simulations, the perimeter-adjusted metric has an absolute error of less than 0.05 mm/year. Nevertheless, for both metrics, errors tend to increase for increasing growth rates. Figure 6 shows representative growth simulation data, and Figure 7 shows plots of the mean per-eye absolute estimation errors as a function of several baseline lesion characteristics.

## Discussion

The analyses of this study suggest that the perimeter-adjusted metric more accurately measures length-type GA growth rate compared with metrics that use the square-root transformation (i.e., the effective radius or square-root-of-area metrics). These findings complement those of Shen et al,^11^ who reported that the perimeter-adjusted metric was statistically independent of lesion perimeter, circularity, and focality, per evaluation on the AREDS dataset. Future studies on larger patient cohorts are needed to understand more fully the clinical implications of the improved accuracy of the perimeter-adjusted metric. However, from Figure 5, we can note that the perimeter-adjusted metric shows lower errors and variances than the effective radius metric. This property, combined with its decreased dependency on lesion geometry (Fig 7), suggests that the perimeter-adjusted metric would be better powered to detect reductions in GA growth rates in clinical trials of GA therapeutics, a point that was explored by Shen et al^11^ (Table 5 of their article). However, we expect that the perimeter-adjusted metric will have its greatest practical significance in clinical trials wherein the therapeutic effect size is modest, the number of eyes enrolled is small (e.g., phase 1), the participants are not well randomized, the lesions are not representative of a more general population of interest (e.g., because of a particular geometry-related inclusion criterion), or a combination thereof. Beyond clinical trials, the improved accuracy of the perimeter-adjusted approach may prove important in scientific studies of GA growth, such as examining correlations between GA growth and choriocapillaris impairments,^15^, ^16^, ^17^, ^18^ which often use smaller convenience samples. In any case, because the square-root and perimeter-adjusted metrics are comparable in terms of computational difficulty (equation 1 vs. equation 4), the barrier to using the perimeter-adjusted metric is somewhat lowered.

Based on our analytical derivations and the results from our numerical simulations, the reduced accuracy of square-root-transformed metrics seems to be a consequence of their relatively strong dependence on lesion focality and circularity, both of which affect the lesion’s perimeter-to-area ratio. Despite its superior performance, the perimeter-adjusted metric, like the square-root-transformed metrics, shows a trend of decreasing accuracy with increasing growth rate. For the perimeter-adjusted metric, this decrease in accuracy is likely the result of (1) the increased intrafocus and interfoci merging that occurs at increased growth rates and (2) an exacerbation of the effects of anisotropic growth. In particular, larger anisotropic growths will cause greater increases in the follow-up lesion’s perimeter-to-area ratio, which can skew the estimation. Fortunately, most of the GA growth rates encountered in clinical data are sufficiently slow such that severe failures of the perimeter adjusted growth rate are relatively uncommon.

The biological interpretation of GA growth metrics afforded by our growth modeling framework may help to guide the selection of the type of growth rate metrics that are used for clinical trials of GA therapeutics. For example, because length-type GA growth rates can be viewed as position-time averages of the growth field (equation SI-3, Appendix 1), they are most appropriate when lesion geometry is not of interest. In particular, we argue that this is often the case when estimating (global) GA therapeutic effects, wherein variations in baseline lesion geometry can obfuscate relevant biology. Indeed, within our growth modeling framework, therapeutic efficacy can be interpreted as a reduction of the values of the growth field. However, scenarios exist in which area-type growth rate metrics are desirable. For example, if predicting visual field loss is of interest, both the lesion geometry (size and location) and the growth field are relevant, and the natural coupling of these factors in area-rate metrics is advantageous.

It is interesting to note that the square-root-transformed metrics tend to overestimate the growth rate, whereas the perimeter-adjusted growth rates tend to underestimate the growth rate. The overestimation of square-root-transformed metrics can be understood as a consequence of the inverse proportionality to the square root of circularity (equation 2) and the $\sqrt{n}$ dependency on focality (equation 3). The underestimation of the perimeter-adjusted growth rates can be understood as a consequence of lesion merging, which censors GA growth (Appendix 3) and anisotropic growth fields, which, as mentioned, increase the follow-up lesion’s perimeter-to-area ratio.

Considering the scatterplots of Figure 5, we see that with increasing follow-up intervals, a trend of improving accuracy for the effective radius metric and a worsening accuracy for the perimeter adjusted metric emerges. The latter is expected and can be understood as a consequence of increased lesion merging (see, for example, merging at the longer intervals in Fig 6). The former is somewhat less expected, but can be understood as a consequence of the increasing circularity and decreasing focality of follow-up lesions in the large growth limit; that is, the follow-up lesions tend to look more like (unifocal) circles at longer follow-up intervals (Fig 6). Moreover, because the follow-up lesions have substantially more area than the baseline lesions, they dominate the contribution of the baseline lesion areas in the difference of square-root-of-areas computation (equation 1). However, an important caveat to this trend is that, in clinically observed GA growth patterns, we expect to see the formation of new lesion foci during the follow-up interval, the probability of which increases with longer follow-up intervals. Such new foci formation would tend to decrease the accuracy of the square-root-transformed metrics. Notably, new foci formation was not modeled in our simulations, a limitation discussed later.

Although the mathematical formulation and numerical implementation of our growth model is somewhat complex, our model’s conceptual underpinning is relatively parsimonious. Indeed, the atrophy front growth model of equation SI-2 (Appendix 1) is arguably the minimal model of an anisotropically evolving margin. Moreover, as noted previously, although independently derived, our growth model naturally leads to a notion of growth rate that is consistent with the previously proposed square-root-transformed and perimeter-adjusted growth rate metrics. We argue that this agreement lends support both to our model and to the use of these growth rate metrics. Nevertheless, given the relative intricacies of our model’s formulation, it is reasonable to ask what advantages it offers compared with more informal or ad hoc statements of the margin-mediated growth hypothesis. In this regard, we believe that this study makes 2 substantive contributions: (1) our model leads to a precise biophysical statement as to what length-type growth rate metrics measure, something that is reasonably nuanced and not a priori evident, particularly for anisotropic lesion growths; and (2) our model allows quantitative assessments of length-type metric accuracies on realistic lesion geometries and growth patterns, something that is not possible with existing formulations. In particular, the accuracies of length-type metrics have been discussed previously only in the context of simple shapes (e.g., circles) and simple growth patterns (e.g., isotropic growth fields or infinitesimal time intervals, Δ*t*).

In this study, we used our atrophy-front growth model as part of a framework to evaluate the accuracies of existing length-type growth rate metrics. In particular, we did not use the atrophy-front growth model as a metric of GA growth, per se. However, in the case of isotropic GA growth that obeys our atrophy front growth model (equation SI-2, Appendix 1), it is possible to invert the atrophy-front model and estimate the ground-truth growth rate using only the initial and follow-up margins. Furthermore, it can be shown (Appendix 10) that for time-invariant isotropic growth fields, such inversion leads to a metric that is an exact estimator for arbitrary lesion geometries. Notably, this approach removes the constraints related to intrafocus (Appendix 3) and interfoci merging that apply to the perimeter-adjusted metric. Moreover, even for anisotropic growth fields, the inversion process, although no longer exact, remains relatively stable. Despite these advantages, we opted not to pursue such a metric in this study for 2 reasons. First, because the simulated GA growth data are generated using the atrophy-front model, it may be misleading to use these data to assess a metric based on this model. Second, the process of inverting equation 3 involves considerably more computation than does the perimeter-adjusted metric, and the performance of the perimeter-adjusted metric is sufficiently accurate under most clinically relevant situations. Nevertheless, because it may have applications in certain scenarios, we have included in Appendix 10 a procedure for computing a length-type metric based on atrophy-front inversion.

This study has a number of limitations that are important to consider. First, the study is simulation based. A simulation-based approach was chosen because it is not possible to evaluate ground truth length-type growth rates (equation SI-3, Appendix 1) using standard clinical GA data. In particular, evaluation of ground truth length-type growth rates requires time-dense growth rate measurements (e.g., monthly GA measurements), which are not available commonly. To mitigate this limitation, we made use of clinically observed baseline lesion geometries and random growth fields producing GA growths having statistical properties approximately matching those of clinically observed data. In future studies, if time-dense GA measurements are available, it may be possible to estimate the growth field from these measurements and then use this estimated growth field in the place of the simulated growth fields. Another limitation of our study is the relatively small number of GA eyes used for our semisimulated analysis. However, we believe that our cohort captures many of the common variations in lesion geometry. Moreover, we were able to augment our dataset by generating multiple (n = 100) follow-up lesion boundaries using different simulated growth fields. We also note that our study is limited in that the reported accuracies must be interpreted within the context of the growth model; that is, the reported accuracies are meaningful to the degree to which this model captures true GA growth physiologic features. At first, this may seem like a serious limitation—and, in some senses, it is—because this model surely does not fully represent true GA growth physiologic features. However, it is important to note that the very use of length-type growth rate metrics assumes some model of GA growth, even if that model is unstated or stated only for simple geometries. For example, the widely used square-root-transformed growth metrics are based on a model of GA growth involving a unifocal circular lesion and isotropic, time-invariant growths. Thus, compared with existing characterizations, our model and assessment represent substantial improvements in capturing the complexities of GA growth. A somewhat related limitation arises from our construction of the random growth fields (Appendix 6). Because the empirical data regarding the spatial statistics of GA growth rates are very limited, our simulated growth field construction largely was ad hoc and was guided only by basic physiologic plausibility (e.g., the requirement of nonnegative growth field values and spatial variations on the scale of the margin perimeter) and global growth statistics (e.g., approximate matching of the mean and standard deviation of Λ to those reported from clinical datasets). Importantly, our random fields did not model drifts in Λ as a function of eccentricity, which is an ongoing area of investigation.^10^^,^^19^^,^^21^^,^^26^, ^27^, ^28^ However, we do not believe that this had any substantial impact on the general trends observed in our study. We also opted to investigate only time-invariant random fields because of the relative scarcity of data on the (local) time dynamics of GA growth rates. Although certainly a limitation, we again suggest that compared with existing characterizations (e.g., isotropic, time-invariant growth fields), our analyses reflect a substantial improvement in physiologic plausibility. A final limitation of our analysis is that we assessed metric accuracy only in the context of existing lesion foci. Although we expect the results largely to be similar for newly appearing lesion foci, we opted to avoid their analysis in this study because (1) their measurement is highly influenced by the precise time between the baseline and follow-up visits at which they first appear^19^ and (2) it has been suggested that the processes governing new foci development may differ from those governing growth of existing foci.^29^, ^30^, ^31^ However, as mentioned previously, we do expect that modeling newly appearing foci will decrease the reported accuracies of the effective radius growth metric, particularly for the 5-year follow-up data.

As a final note, we believe it is useful to draw attention to some additional applications of our growth field formalism that may have usefulness beyond the assessment of growth rate metrics. For example, by using the atrophy-front growth model, the problem of developing predictive models of GA growth is translated into a problem of growth field estimation. This has several attractive features, including that it naturally constrains GA growth prediction within a biologically interpretable model and that such spatial fields can be parametrized directly with en face imaging biomarkers (e.g., choriocapillaris impairment or basal linear or laminar deposits). Alternatively, working in the reverse direction, coupled with optimization frameworks, the growth field estimation could be used to identify en face imaging biomarkers. Although not explored in this article, we believe that these are fruitful directions for future investigations.

## Conclusions

In this study, we showed that length-type GA growth rates can be interpreted as time-position averages of corresponding growth fields. Using simulated and semisimulated growth data, we evaluated the accuracies of square-root-transformed and perimeter-adjusted growth metrics and demonstrated the superiority of perimeter adjustment. Our study provides additional justification for using the perimeter-adjusted metric to measure GA growth rates in clinical trials of GA therapeutics.

## References

[1] Sadda S.R., Guymer R., Holz F.G. (2018). Consensus definition for atrophy associated with age-related macular degeneration on OCT: Classification of Atrophy report 3. Ophthalmology.

[2] Bhutto I., Lutty G. (2012). Understanding age-related macular degeneration (AMD): relationships between the photoreceptor/retinal pigment epithelium/Bruch’s membrane/choriocapillaris complex. Mol Aspects Med.

[3] Bird A.C., Phillips R.L., Hageman G.S. (2014). Geographic atrophy: a histopathological assessment. JAMA Ophthalmol.

[4] Li M., Huisingh C., Messinger J. (2018). Histology of geographic atrophy secondary to age-related macular degeneration: a multilayer approach. Retina.

[5] Jaffe G.J., Westby K., Csaky K.G. (2021). C5 inhibitor Avacincaptad pegol for geographic atrophy due to age-related macular degeneration: a randomized pivotal phase 2/3 trial. Ophthalmology.

[6] Liao D.S., Grossi F.V., El Mehdi D. (2020). Complement C3 inhibitor pegcetacoplan for geographic atrophy secondary to age-related macular degeneration: a randomized phase 2 trial. Ophthalmology.

[7] Schaal K.B., Rosenfeld P.J., Gregori G. (2016). Anatomic clinical trial endpoints for nonexudative age-related macular degeneration. Ophthalmology.

[8] Yehoshua Z., Rosenfeld P.J., Gregori G. (2011). Progression of geographic atrophy in age-related macular degeneration imaged with spectral domain optical coherence tomography. Ophthalmology.

[9] Feuer W.J., Yehoshua Z., Gregori G. (2013). Square root transformation of geographic atrophy area measurements to eliminate dependence of growth rates on baseline lesion measurements: a reanalysis of Age-Related Eye Disease Study report no. 26. JAMA Ophthalmol.

[10] Shen L.L., Sun M., Khetpal S. (2020). Topographic variation of the growth rate of geographic atrophy in nonexudative age-related macular degeneration: a systematic review and meta-analysis. Invest Ophthalmol Vis Sci.

[11] Shen L.L., Sun M., Ahluwalia A. (2021). Geographic atrophy growth is strongly related to lesion perimeter: unifying effects of lesion area, number, and circularity on growth. Ophthalmol Retina.

[12] Fleckenstein M., Mitchell P., Freund K.B. (2018). The progression of geographic atrophy secondary to age-related macular degeneration. Ophthalmology.

[13] Pfau M., Lindner M., Goerdt L. (2019). Prognostic value of shape-descriptive factors for the progression of geographic atrophy secondary to age-related macular degeneration. Retina.

[14] Domalpally A., Danis R.P., White J. (2013). Circularity index as a risk factor for progression of geographic atrophy. Ophthalmology.

[15] Moult E.M., Alibhai A.Y., Lee B. (2020). A framework for multiscale quantitation of relationships between choriocapillaris flow impairment and geographic atrophy growth. Am J Ophthalmol.

[16] Thulliez M., Zhang Q., Shi Y. (2019). Correlations between choriocapillaris flow deficits around geographic atrophy and enlargement rates based on swept-source OCT imaging. Ophthalmol Retina.

[17] Nassisi M., Baghdasaryan E., Borrelli E. (2019). Choriocapillaris flow impairment surrounding geographic atrophy correlates with disease progression. PloS One.

[18] Alagorie A.R., Nassisi M., Verma A. (2020). Relationship between proximity of choriocapillaris flow deficits and enlargement rate of geographic atrophy. Graefes Arch Clin Exp Ophthalmol.

[19] Moult E.M., Hwang Y., Shi Y. (2021). Growth modeling for quantitative, spatially-resolved geographic atrophy lesion kinetics. Transl Vis Sci Technol.

[20] Moult E.M., Shi Y., Zhang Q. (2021). Analysis of correlations between local geographic atrophy growth rates and local OCT angiography-measured choriocapillaris flow deficits. Biomed Opt Express.

[21] Uji A., Nittala M.G., Hariri A. (2019). Directional kinetics analysis of the progression of geographic atrophy. Graefes Arch Clin Exp Ophthalmol.

[22] Yezzi A.J., Prince J.L. (2003). An Eulerian PDE approach for computing tissue thickness. IEEE Trans Med Imaging.

[23] Hanus J., Anderson C., Wang S. (2015). RPE necroptosis in response to oxidative stress and in AMD. Ageing Res Rev.

[24] Gallagher-Colombo S., Maminishkis A., Tate S. (2010). Modulation of MCT3 expression during wound healing of the retinal pigment epithelium. Invest Ophthalmol Vis Sci.

[25] Farouki R.T., Neff C.A. (1990). Analytic properties of plane offset curves. Comput Aided Geom Des.

[26] Mauschitz M.M., Fonseca S., Chang P. (2012). Topography of geographic atrophy in age-related macular degeneration. Invest Ophthalmol Vis Sci.

[27] Sayegh R.G., Sacu S., Dunavölgyi R. (2017). Geographic atrophy and foveal-sparing changes related to visual acuity in patients with dry age-related macular degeneration over time. Am J Ophthalmol.

[28] Lindner M., Böker A., Mauschitz M.M. (2015). Directional kinetics of geographic atrophy progression in age-related macular degeneration with foveal sparing. Ophthalmology.

[29] Keenan T.D. (2021). Re: Jaffe et al.: C5 inhibitor avacincaptad pegol for geographic atrophy due to age-related macular degeneration: a randomized pivotal phase 2/3 trial. Ophthalmology.

[30] Grassmann F., Harsch S., Brandl C. (2019). Assessment of novel genome-wide significant gene loci and lesion growth in geographic atrophy secondary to age-related macular degeneration. JAMA Ophthalmol.

[31] Gorin M.B. (2019). When genetics can point researchers and clinicians in new directions. JAMA Ophthalmol.

