# Supplementary material for: Comparing Accuracies of Length-Type Geographic Atrophy Growth Rate Metrics Using Atrophy-Front Growth Modeling
Source: Ophthalmol Sci. 2022 Apr 14;2(3):100156. doi: 10.1016/j.xops.2022.100156 (PMC9560575; doi:10.1016/j.xops.2022.100156)
Supplement: Appendix 1 [file mmc1.pdf]

Supplement I: The Atrophy-Front Model and its Connection to Length-Type Growth Rate Metrics

I.i The Atrophy-Front Model of GA Growth

Letting $\Omega \subset \mathbb{R}^2$ describe the domain of fundus positions, we denote the GA region and GA margin at time t by $G(t) \subset \Omega$ and $\partial G(t) \subset \Omega$, respectively. Because GA growth is characterized by an enlarging region of atrophy, and not the transport of any material, it is conceptually and computationally convenient to describe GA growth using an implicit representation of the GA margin (rather than an explicitly parameterized representation). In particular, we let $G(t) = \{\boldsymbol{x} \in \Omega : \phi(\boldsymbol{x}, t) \leq 0\}$ and $\partial G(t) = \{\boldsymbol{x} \in \Omega : \phi(\boldsymbol{x}, t) = 0\}$, where $\phi(\boldsymbol{x}, t) : \Omega \times \mathbb{R} \to \mathbb{R}$ is initialized as the signed-distance level set function.[1] In this implicit representation, our GA growth model takes the general form (cf. Eq. 4.4 of ref.[2]):

$$\partial_t \phi(\boldsymbol{x}, t) + \mu(\phi, v, \boldsymbol{x}, t) ||\nabla \phi(\boldsymbol{x}, t)|| = 0 \quad \text{(SI-1)}$$

where ∂_t is the partial derivative in time, μ is a function determining the rate at which the margin expands, $v(\boldsymbol{x}, t)$ is the growth field, which determines the geometry-*independent* rate at which the lesion margin expands outward, and ∇ is the (spatial) gradient operator. While we have previously[3] provided rationale for a curvature term in the context of local growth rate measurements, its practical effect on global lesion growth is small, and, for simplicity, throughout this manuscript we neglect it. In particular, in this paper, we assume that $\mu(\phi, v, \boldsymbol{x}, t) = v(\boldsymbol{x}, t)$, which simplifies Eq. SI-1 to:

$$\partial_t \phi(\boldsymbol{x}, t) + v(\boldsymbol{x}, t) ||\nabla \phi(\boldsymbol{x}, t)|| = 0 \quad \text{(SI-2)}$$

Mathematically, we can identify the growth field $v(\boldsymbol{x}, t)$ as the speed associated with an advective field. Physiologically, we can identify the growth field as capturing those elements of the chorioretinal milieu that influence GA growth rate.

I.ii GA Growth Metrics as Position-Time Growth Field Averages

In the atrophy-front growth model of Eq. SI-2, the baseline lesion geometry and the growth field $v(\boldsymbol{x}, t)$ are the sole determinants of GA growth. It therefore follows that any geometry-independent growth

metric should be derivable from $v(\boldsymbol{x},t)$, at least within the context of the atrophy-front growth model. This makes $v(\boldsymbol{x},t)$ a natural parameter from which to interpret length-type growth rates. Moreover, $v(\boldsymbol{x},t)$ has units of distance per time, matching those of length-type metrics. However, because $v(\boldsymbol{x},t)$ is dependent on both fundus position, $\boldsymbol{x}$, and time, t, it is a *local* descriptor of lesion growth, and, consequently, is not itself a *global* GA growth metric. Nevertheless, it is natural to define a corresponding global growth rate, Λ, as a position-time average of the growth field values along the lesion margin during growth, which can be expressed mathematically as:

$$\Lambda \equiv \frac{1}{\Delta t}\int_{t_b}^{t_f}\frac{1}{P(t)}\oint_{\partial G(t)} v(\boldsymbol{x},t)\,d\ell\,dt \qquad \text{(SI-3)}$$

where t_b and t_f are the times of the baseline and follow-up visits, $\Delta t = t_f - t_b$ is the intervisit time, and $P(t)$ is the lesion perimeter at time t. Eq. SI-3 is a key definition, and, in words, specifies length-type growth rate as averages of the growth field values coincident with the GA margin during its growth. Alternatively stated, Λ is the (position-time) average speed at which the entirety of the margin is locally expanding. Note that for time-invariant, isotropic growth fields, Eq. SI-3 simplifies to $\Lambda = v$.

For the purposes of this paper, we take Λ as the ground truth global length-type growth rate—that is, Λ is the parameter to be estimated, and the parameter against which growth rate metrics are assessed. Accordingly, we use the notation $\widehat{\Lambda}$ to denote estimators (metrics) of Λ. Although Eq. SI-3 is not the only possible definition of a global metric of $v(\boldsymbol{x},t)$, it intuitively quantifies the interactions between the lesion margin and the growth field and, as described in the main text, leads to results that agree with existing length-type GA growth rates in simple geometries.

References

1. Osher S, Sethian JA. Fronts propagating with curvature-dependent speed: Algorithms based on Hamilton-Jacobi formulations. *Journal of Computational Physics* 1988;79:12-49.
2. Osher S, Fedkiw R. *Level set methods and dynamic implicit surfaces*: Springer Science & Business Media; 2006.

3. Moult EM, Alibhai AY, Lee B, et al. A Framework for Multiscale Quantitation of Relationships Between Choriocapillaris Flow Impairment and Geographic Atrophy Growth. *American Journal of Ophthalmology* 2019.
