# Supplementary material for: Comparing Accuracies of Length-Type Geographic Atrophy Growth Rate Metrics Using Atrophy-Front Growth Modeling
Source: Ophthalmol Sci. 2022 Apr 14;2(3):100156. doi: 10.1016/j.xops.2022.100156 (PMC9560575; doi:10.1016/j.xops.2022.100156)
Supplement: Appendix 2 [file mmc2.pdf]

Supplement II: Effect of Circularity on the Effective Radius Metric

Consider the case of a unifocal lesion $G(t)$ that is expanding isotropically outward at a rate of v mm/year. With baseline and follow-up times, t_b and $t_f = t_b + \Delta t$, respectively, and assuming that $v\Delta t \min(\kappa(\boldsymbol{x})) > -1$ for $\boldsymbol{x} \in \partial G(t_b)$, where, $\partial G(t_b)$ is the GA margin at baseline, and $\kappa(\boldsymbol{x})$ is the margin curvature at a margin point $\boldsymbol{x}$, we have that, per Eq. 19 and Eq. 24 of Farouki and Neff:[1]

$$P(t_f) = P(t_b) + 2\pi v\Delta t \quad \text{(SII-1)}$$

$$A(t_f) - A(t_b) = \frac{1}{2}\left(P(t_f) + P(t_b)\right) v\Delta t \quad \text{(SII-2)}$$

where $A(t)$ and $P(t)$ are the lesion area and perimeter at time t, respectively, and $v\Delta t$ corresponds to the margin offset. Combining Eqs. SII-1 and SII-2 yields:

$$A(t_f) = A(t_b) + \pi(v\Delta t)^2 + P(t_b)v\Delta t \quad \text{(SII-3)}$$

Thus, per Eq. 1 (main text), the effective radius metric, $\widehat{\Lambda}_{\mathrm{ER}}$, is:

$$\widehat{\Lambda}_{\mathrm{ER}} = \frac{\sqrt{A(t_f)} - \sqrt{A(t_b)}}{\sqrt{\pi}\Delta t} = \frac{\sqrt{A(t_b) + \pi(v\Delta t)^2 + P(t_b)v\Delta t} - \sqrt{A(t_b)}}{\sqrt{\pi}\Delta t} \quad \text{(SII-4)}$$

where $A(t)$ and $P(t)$ are the lesion area and perimeter at time t, respectively. Note that we can have:

$$\sqrt{A(t_b) + \pi(v\Delta t)^2 + P(t_b)v\Delta t} = \sqrt{A(t_b)} \cdot \sqrt{1 + \frac{\pi(v\Delta t)^2 + P(t_b)v\Delta t}{A(t_b)}} \quad \text{(SII-5)}$$

Because $\sqrt{1+y} = 1 + \frac{1}{2}y + \mathcal{O}(y^2)$, for $\pi(v\Delta t)^2 + P(t_b)(v\Delta t) \ll A(t_b)$, we can re-write Eq. SII-5 to first order as:

$$\sqrt{A(t_b) + \pi(v\Delta t)^2 + P(t_b)v\Delta t} \approx \sqrt{A(t_b)}\left(1 + \frac{P(t_b)v\Delta t}{2A(t_b)}\right) \quad \text{(SII-6)}$$

Thus, in this small-growth regime, substituting Eq. SII-6 into Eq. SII-4 yields:

$$\widehat{\Lambda}_{ER} \approx \frac{P(t_b)v}{2\sqrt{\pi A(t_b)}} \quad \text{(SII-7)}$$

Finally, defining circularity as $\mathrm{circ}(G(t)) \equiv 4\pi A(t)/P^2(t)$ we have that:

$$\widehat{\Lambda}_{ER} \approx \frac{v}{\sqrt{\mathrm{circ}\left(G(t_b)\right)}} \tag{SII-8}$$

References

1. Farouki RT, Neff CA. Analytic properties of plane offset curves. *Computer aided geometric design* 1990;7:83-99.
