# Supplementary material for: Comparing Accuracies of Length-Type Geographic Atrophy Growth Rate Metrics Using Atrophy-Front Growth Modeling
Source: Ophthalmol Sci. 2022 Apr 14;2(3):100156. doi: 10.1016/j.xops.2022.100156 (PMC9560575; doi:10.1016/j.xops.2022.100156)
Supplement: Appendix 3 [file mmc3.pdf]

Supplement III: Intrafocus Merging and the Perimeter Adjusted Metric

For the perimeter adjusted growth rate metric, $\widehat{\Lambda}_{\mathrm{PA}}$, defined in Eq. 4, it can be It can be shown[1] that $\widehat{\Lambda}_{\mathrm{PA}}$ is an exact estimator of the true length-type growth rate, Λ, defined in Eq. SI-3 of Supplement I, for 'small' lesion growth. Precisely, $\widehat{\Lambda}_{\mathrm{PA}} = \Lambda$ if:

$$v\Delta t \cdot \min_{\boldsymbol{x}} \kappa(\boldsymbol{x}) > -1 \qquad \text{for} \quad \boldsymbol{x} \in \partial G(t_b) \qquad \text{(SIII-1)}$$

where v is the rate of lesion enlargement (i.e., the value of the isotropic growth field), Δt is the time between the baseline and follow-up visits, $\partial G(t_b)$ is the GA margin at baseline, and $\kappa(\boldsymbol{x})$ is the margin curvature at a margin point $\boldsymbol{x}$. To understand the connection between intrafocus merging and the condition of Eq. SIII-1, it is helpful to consider the simulated margin segment of Figure S1. In this figure, the margin points $\boldsymbol{x}_1$ and $\boldsymbol{x}_3$ correspond to concave margin segments (i.e., $\kappa(\boldsymbol{x}_1), \kappa(\boldsymbol{x}_3) < 0$, where $\kappa(\boldsymbol{x})$ is the curvature at margin point $\boldsymbol{x}$) and the margin point $\boldsymbol{x}_2$ corresponds to a convex margin segment (i.e., $\kappa(\boldsymbol{x}_2) > 0$). Note also that margin at $\boldsymbol{x}_1$ is 'less curved' than at $\boldsymbol{x}_3$ (i.e., $|\kappa(\boldsymbol{x}_1)| < |\kappa(\boldsymbol{x}_3)|$); in particular, $\kappa(\boldsymbol{x}_1)$ = -8 mm^{-1} and $\kappa(\boldsymbol{x}_3)$ = -32 mm^{-1}. After 1 year of growth at v = 0.1 mm/year, as shown in Figure S1.a, $v\Delta t$ = 0.1 mm, we have that $v\Delta t \cdot \kappa(\boldsymbol{x}_1) = -0.8 > -1$ and $v\Delta t \cdot \kappa(\boldsymbol{x}_3) = -3.2 < -1$. Thus, $\boldsymbol{x}_1$ satisfies Eq. SIII-1, but $\boldsymbol{x}_3$ does not. Correspondingly, as shown in Figure S1.a, there is intrafocus merging associated with $\boldsymbol{x}_3$, but not with $\boldsymbol{x}_1$. After 2 years of growth at v = 0.1 mm/year, as shown in Figure S1.b, $v\Delta t$ = 0.2 mm, and we have that $v\Delta t \cdot \kappa(\boldsymbol{x}_1) = -1.6 < -1$ and $v\Delta t \cdot \kappa(\boldsymbol{x}_3) = -6.4 < -1$. Thus, neither $\boldsymbol{x}_1$ nor $\boldsymbol{x}_3$ satisfy Eq. SIII-1, and both points are associated with intrafocus merging. It is this 'censoring' of GA growth, due to intrafocus merging, that leads to the condition of Eq. SIII-1. Note that, in contrast, since $\kappa(\boldsymbol{x}_2)$ > 0, $\boldsymbol{x}_2$ satisfies Eq. SIII-1 for any growth rate and follow-up time—convex lesion segments are not involved in intrafocus merging.

References

1. Farouki RT, Neff CA. Analytic properties of plane offset curves. *Computer aided geometric design* 1990;7:83-99.
