# Supplementary material for: Comparing Accuracies of Length-Type Geographic Atrophy Growth Rate Metrics Using Atrophy-Front Growth Modeling
Source: Ophthalmol Sci. 2022 Apr 14;2(3):100156. doi: 10.1016/j.xops.2022.100156 (PMC9560575; doi:10.1016/j.xops.2022.100156)
Supplement: Appendix 4 [file mmc4.pdf]

Supplement IV: Numerical Implementation of Lesion Growth Simulation and Analysis

Baseline lesions were evolved in time by numerically solving Eq. SI-2 using an open source implementation of the fast marching method.[1] Prior to performing the fast marching, baseline lesion margins, described in Supplements V and VII, were upsampled 3× to improve accuracy (note that the random growth field data, described in Supplements VI and VIII, were generated on a 3000 pixel × 3000 pixel grid, 3× denser in each dimension than that of the lesion data, and therefore were not upsampled). To compute Λ as defined in Eq. SI-3 (Supplement I), the (spatial) mean of the growth field v was computed along the lesion margin at 10 equally spaced time points in the range $[t_f, t_b]$, and these 10 means were then (time) averaged (see Figure 1). To compute the mean of the growth field v along the lesion margin, the margin perimeter was sampled at equally spaced (in arc length) 6 µm increments, with sampled v values computed via bilinear interpolation.

References

1. Peyre G. Toolbox Fast Marching MATLAB Central File Exchange. 2021.
