# Supplementary material for: Comparing Accuracies of Length-Type Geographic Atrophy Growth Rate Metrics Using Atrophy-Front Growth Modeling
Source: Ophthalmol Sci. 2022 Apr 14;2(3):100156. doi: 10.1016/j.xops.2022.100156 (PMC9560575; doi:10.1016/j.xops.2022.100156)
Supplement: Appendix 5 [file mmc5.pdf]

Supplement V: Details of Simulated GA Growth Data for Section 2.2.2

For the lesion growth patterns of Section 2.2.2, both the baseline GA geometries, $G(t_b)$, and growth fields, $v(\boldsymbol{x})$, were simulated. Baseline lesion geometries were constructed so that baseline lesion areas, $A(t_b)$, were 6 mm^2. Values of $v(\boldsymbol{x})$ were constructed to yield the desired Λ, which was varied between 0.05 mm/year and 0.70 mm/year. The specifications of $G(t_b)$ and $v(\boldsymbol{x})$ are provided below for each of the configurations. For the purposes of computation (Supplement IV), all lesions were embedded in a synthetic 12 mm × 12 mm field-of-view comprised of 1000 pixels × 1000 pixels. As discussed in Supplement VII, the 12 µm × 12 µm pixel dimension of this grid matches that used for Section 2.2.3.

Configuration 1: $G(t_b)$ was constructed as a unifocal circular lesion of area $A(t_b)$. The growth field was constant everywhere: $v(\boldsymbol{x})$ = Λ mm/year.

Configuration 2: $G(t_b)$ was constructed as a unifocal elliptical lesion having an eccentricity of 0.8 and an area $A(t_b)$. The growth field was constant everywhere: $v(\boldsymbol{x})$ = Λ mm/year.

Configuration 3: $G(t_b)$ was constructed by geometrically 'subtracting' three parabolas from a unifocal circular lesion. Each parabola had a focus of 10 µm with its vertex located equidistant from the circle's center and margin. The radius of the circle was then chosen so that the area of resultant shape was $A(t_b)$. The growth field was constant everywhere: $v(\boldsymbol{x})$ = Λ mm/year.

Configuration 4: $G(t_b)$ was comprised of a unifocal circular lesion of area $A(t_b)$. The v field was constructed as to have a 'notched' variation with respect to $\theta = \operatorname{atan2}(\boldsymbol{x_2}, \boldsymbol{x_1}) \in [0, 2\pi)$, the azimuthal angle in a polar coordinate system. In particular:

$$v(\theta) = \begin{cases} v_o & \text{for} \quad \theta \notin \mathcal{N} \\ v_1 & \text{for} \quad \theta \in \mathcal{N} \end{cases}$$

where $\mathcal{N} = \bigcup_{i=1}^{3} \left\{\theta \in [\varphi_i - \frac{\Delta\theta}{2}, \varphi_i + \frac{\Delta\theta}{2}]\right\}$, where $\varphi_i = \frac{2\pi i}{3}$, $\Delta\theta = \frac{\pi}{9}$, and the value of v_o was fixed at 0.05 mm/year. The value of v_1 was computed to yield the desired Λ.

Configuration 5: $G(t_b)$ was comprised of two identical circular lesions, each of area $\frac{1}{2}A(t_b)$. The growth field was constant everywhere: $v(\boldsymbol{x})$ = Λ . The lesions were positioned so that there was no merging over the considered 1-year follow-up interval.

Configuration 6: $G(t_b)$ was comprised of circular lesions, one of area $\frac{1}{8}A(t_b)$ and the other of area $\frac{7}{8}A(t_b)$ The growth field was constant everywhere: $v(\boldsymbol{x})$ = Λ. The lesions were positioned so that there was no merging over the considered 1-year follow-up interval.

Configuration 7: $G(t_b)$ was comprised of two identical circular lesions, each of area $\frac{1}{2}A(t_b)$. The growth field $v(\boldsymbol{x})$ was constructed to be v_1 in the vicinity of one lesion, and $2v_1$ in the vicinity of the other. The value of v_1 was computed to yield the desired Λ, and growth field areas in regions outside of the follow-up margins were computed via Laplacian interpolation. The lesions were positioned so that there was no merging over the considered 1-year follow-up interval.

Configuration 8: $G(t_b)$ was comprised of two identical circular lesions, each of area $\frac{1}{2}A(t_b)$. The circular lesions were positioned so that, at baseline, the minimum distance between margin points was $v(\boldsymbol{x})\Delta t/8$ **,** which ensures lesion merging during the follow-up interval. The growth field was constant everywhere: $v(\boldsymbol{x})$ = Λ.
