# Supplementary material for: Comparing Accuracies of Length-Type Geographic Atrophy Growth Rate Metrics Using Atrophy-Front Growth Modeling
Source: Ophthalmol Sci. 2022 Apr 14;2(3):100156. doi: 10.1016/j.xops.2022.100156 (PMC9560575; doi:10.1016/j.xops.2022.100156)
Supplement: Appendix 6 [file mmc6.pdf]

Supplement VI: Simulations with Observed Lesion Geometries and Random Growth Fields

As noted in the text, our aim was to generate growth fields, $v(\boldsymbol{x})$, having: (a) variations in global growth rates; (b) variations in local growth rates; and, (c) growth rate statistics approximating those of clinically reported data. To achieve these requirements, our simulated growth fields were formed as:

$$v(\boldsymbol{x}) = H(Y(c_1) + c_2F(\boldsymbol{x}) + c_3) \qquad \text{(SVI-1)}$$

where H is the Heaviside function, $Y(c_1) \sim \exp(c_1)$ is a scalar-valued exponential random variable, $F(\boldsymbol{x})$ is a Gaussian random field with a Matérn covariance model, and c_1, c_2, and c_3, are positive constant scalars. With reference to Eq. SVI-1, note that Y models global (i.e., between-eye) growth rate variations, while F models local (i.e., within-eye) growth rate variations. The Heaviside function H ensures that all growth rates are positive. In the Matérn model, the covariance C between growth rates at locations separated by r mm is:[1]

$$C(r) = \frac{2^{1-\gamma}}{\Gamma(\gamma)}\left(\sqrt{2\gamma}\frac{r}{\rho}\right)^{\gamma} K_{\gamma}\left(\sqrt{2\gamma}\frac{r}{\rho}\right) \qquad \text{(SVI-2)}$$

where γ is a smoothness parameter, ρ determines the spatial scale of correlations, and K_γ is a modified Bessel function of the second kind (note that γ of Eq. SVI-2 is typically denoted by ν; however, we have avoided this notation due to potential confusion with our notation for the growth field, v). Examples of random fields generated via Eqs. SVI-1 and SVI-2 are shown in Figure S2. Intuitively, larger values of γ correspond to smoother random fields, and larger values of ρ correspond to stronger spatial correlations (i.e., a tendency for growth rates at nearby positions to be more similar). Because there is at present limited empirical data regarding the spatial statistics of GA lesion growth, for all our simulations we took the convenience values of $\gamma = 1$ and $\rho = 1$ mm, which generate subjectively plausible variations in local GA growths over the distance scales of typical GA margins. To approximately match the mean (μ_Λ) and standard deviation (σ_Λ) of the resulting global growth rates to those of the 1-year perimeter adjusted growth rates of the AREDS dataset,[2] namely $\mu_\Lambda \approx \sigma_\Lambda \approx 0.1$ mm/year, we set $c_1 = \sqrt{2}/\mu_\Lambda$, $c_2 = \mu_\Lambda/\sqrt{2}$, and

$c_3 = \mu_\Lambda\left(1 - 1/\sqrt{2}\right)$. All random field simulations were generated in R (version 3.6.3; R Foundation for Statistical Computing, Austria) using the package 'RandomFields' (Supplement VIII).[3]

As described in the main text, for each of the 38 lesions margins of this study (see Supplement VII for details of baseline GA lesion data), which we denote $\partial G_i(t_b)$, for $1 \le i \le 38$, we generated 100 random fields, which we denote by $v_i^j(\boldsymbol{x})$, $1 \le j \le 100$. Then, for each baseline lesion margin $\partial G_i(t_b)$, a simulated follow-up lesion margin $\partial G_i^j\left(t_f\right)$ was computed using Eq. SI-2 (see Supplement I). Thus, our complete evaluation set was comprised of 3800 baseline and follow-up GA margin pairs: $\left\{\left(\partial G_i(t_b), \partial G_i^j\left(t_f\right)\right): 1 \le i \le 38, 1 \le j \le 100\right\}$.

References

1. Gelfand AE, Diggle P, Guttorp P, Fuentes M. *Handbook of spatial statistics*: CRC press; 2010.
2. Shen LL, Sun M, Ahluwalia A, Young BK, Park MM, Del Priore LV. Geographic atrophy growth is strongly related to lesion perimeter: unifying effects of lesion area, number, and circularity on growth. *Ophthalmology Retina* 2020.
3. Schlather M, Malinowski A, Menck PJ, Oesting M, Strokorb K. Analysis, simulation and prediction of multivariate random fields with package random fields. *Journal of Statistical Software* 2015;63:1-25.
