# Supplementary material for: Comparing Accuracies of Length-Type Geographic Atrophy Growth Rate Metrics Using Atrophy-Front Growth Modeling
Source: Ophthalmol Sci. 2022 Apr 14;2(3):100156. doi: 10.1016/j.xops.2022.100156 (PMC9560575; doi:10.1016/j.xops.2022.100156)
Supplement: Appendix 7 [file mmc7.pdf]

Supplement VII: Details of Observed GA Growth Data for Section 2.2.3

For the numerical experiments of Section 2.2.3, baseline lesion geometries were extracted from 6 mm × 6 mm *en face* OCT images. These *en face* OCT images were comprised of 500 A-scans per B-scan and 500 B-scans per volume, corresponding to a 12 µm × 12 µm pixel dimension. The lesions were embedded in a simulated 12 mm × 12 mm field-of-view comprised of 1000 pixels × 1000 pixels, which has a matching 12 µm × 12 µm pixel dimension. For the purposes of simulation, in the process of embedding, the lesions were centered on the field-of-view. This centering does not affect the ensemble of resulting growth patterns because, as described in the main text, the random fields used in this study had no spatial trend (i.e., drift).
