# Supplementary material for: Comparing Accuracies of Length-Type Geographic Atrophy Growth Rate Metrics Using Atrophy-Front Growth Modeling
Source: Ophthalmol Sci. 2022 Apr 14;2(3):100156. doi: 10.1016/j.xops.2022.100156 (PMC9560575; doi:10.1016/j.xops.2022.100156)
Supplement: Appendix 8 [file mmc8.pdf]

Supplement VIII: Details of Random Field Generation

As described in Section 2.2.3, random growth fields were generated using the R (version 3.6.3; R Foundation for Statistical Computing, Austria) package ‘RandomFields’.[1] In particular, Gaussian random fields with Matérn covariance models were generated using the ‘RMmatern’ and ‘RFsimulate’ functions over a 12 mm × 12 mm field-of-view comprised of 3000 pixels × 3000 pixels. Note that the sampling for the random fields was 3× denser in each dimension than that for lesion geometry (Supplement VII). This higher sampling density was used to avoid upsampling the random field during the lesion growth simulation, during which the lesion margins are upsampled 3×, as described in Supplement IV.

References

1. Schlather M, Malinowski A, Menck PJ, Oesting M, Strokorb K. Analysis, simulation and prediction of multivariate random fields with package random fields. *Journal of Statistical Software* 2015;63:1-25.
