# Supplementary material for: Comparing Accuracies of Length-Type Geographic Atrophy Growth Rate Metrics Using Atrophy-Front Growth Modeling
Source: Ophthalmol Sci. 2022 Apr 14;2(3):100156. doi: 10.1016/j.xops.2022.100156 (PMC9560575; doi:10.1016/j.xops.2022.100156)
Supplement: Appendix 9 [file mmc9.pdf]

Supplement IX: Gini-Weighted Focality

From the margin-mediated growth hypothesis it is reasonable to expect that, due to their larger perimeters, multifocal lesions will have faster area-type growth rates than unifocal lesions of the same area. Thus, the focality can be an interesting covariate to consider when studying GA growth dynamics. However, being an integer measure, focality does not differentiate between different partitions of lesion area/perimeter. For example, at least for some purposes, it seems reasonable to treat a bifocal lesion with two, equal 3 mm^2 foci differently than a bifocal lesion with two foci, one of which is 5.9 mm^2 and the other of which is 0.1 mm^2. To capture such differences, it is natural to introduce a weighted measure of focality that incorporates some notion of equality. In this paper, we adopt a weighting based on the Gini coefficient, a classical measure of inequality, commonly used in economics and other fields.[1] In particular, letting n denote the number of lesion foci (i.e., the focality), we define the Gini-weighted focality, n_G, as:

$$n_G = 1 + (n-1)(1-G) \qquad \text{(SVII-1)}$$

where G is the normalized Gini index with respect to the foci perimeters:

$$G = \begin{cases} \dfrac{n}{n-1} \cdot \dfrac{\sum_{i=1}^{n} \sum_{j=1}^{n} |P_i(t_b) - P(t_b)|}{2n \sum_{k=1}^{n} P_k(t_b)} & \text{for} \quad n > 1 \\ 1 & \text{for} \quad n = 1 \end{cases} \qquad \text{(SVII-2)}$$

and $P_m(t_b)$ is the baseline area of the *m*-th lesion focus. Note that G ranges between 0 and 1, where the former corresponds to foci with identical perimeters and the latter corresponds to one focus having no perimeter (i.e., the unifocal case). Thus, if the lesion perimeter is equally distributed amongst the foci, $n_G = n$, and if there is unequal distribution, $n_G < n$. Figure S3 illustrates the Gini-weighted focality for different bifocal lesions.

References

1. Sen A, Sen MA, Amartya S, Foster JE, Foster JE. *On economic inequality*: Oxford university press; 1997.
