# Supplementary material for: Comparing Accuracies of Length-Type Geographic Atrophy Growth Rate Metrics Using Atrophy-Front Growth Modeling
Source: Ophthalmol Sci. 2022 Apr 14;2(3):100156. doi: 10.1016/j.xops.2022.100156 (PMC9560575; doi:10.1016/j.xops.2022.100156)
Supplement: Appendix 10 [file mmc10.pdf]

Supplement X: An Atrophy-Front Inversion Global Length-Type Growth Rate Metric

The errors in $\widehat{\Lambda}_{\mathrm{PA}}$ due to intra- and inter-lesion merging can be, in situations, avoided by using what we term the atrophy-front arrival time growth metric, $\widehat{\Lambda}_{\mathrm{AFAT}}$. Assuming the growth model of Eq. SI-2 (Supplement I), $\widehat{\Lambda}_{\mathrm{AFAT}}$ is an exact estimator of the ground truth growth rate Λ (Eq. SI-3, Supplement I) for any lesion geometry and any isotropic growth field, v.

Consider the case of a lesion $G(t)$, with margin $\partial G(t)$, that is expanding isotropically outward at a rate of v mm/year. Suppose that $\partial G(t)$ is observed at baseline and follow-up times, t_b and $t_f = t_b + \Delta t$, respectively. Then, we can compute $\widehat{\Lambda}_{\mathrm{AFAT}}$ by first solving:

$$\partial_t \phi_\lambda(\boldsymbol{x}, t) + \lambda || \nabla \phi_\lambda(\boldsymbol{x}, t) || = 0 \tag{SX-1}$$

where $\phi_\lambda(\boldsymbol{x}, t_b)$ is the signed-distance function representation of $\partial G(t_b)$ and $\lambda > 0$ is some initial 'guess' of the true growth rate, v. Thus, for any follow-up margin point $\boldsymbol{x}_f \in \partial G(t_f)$, the time, $T_\lambda(\boldsymbol{x}_f)$, at which the zero level set of ϕ_λ is coincident with $\boldsymbol{x}_f$ is recorded. Since $T_\lambda(\boldsymbol{x}_f)\lambda = T_v(\boldsymbol{x}_f)v$, and, by assumption $T_v(\boldsymbol{x}_f) = \Delta t$, we have that:

$$\widehat{\Lambda}_{\mathrm{AFAT}} = \frac{T_\lambda(\boldsymbol{x}_f)\lambda}{\Delta t} = v = \Lambda \tag{SX-2}$$

Thus, in the case of the isotropic growth described by Eq. SI-2 (Supplement I), $\widehat{\Lambda}_{\mathrm{AFAT}} = \Lambda$ for any lesion geometry. For anisotropic growths, we can define:

$$\widehat{\Lambda}_{\mathrm{AFAT}} \equiv \frac{1}{P(t_f)} \oint_{\partial G(t_f)} \frac{T_\lambda(\boldsymbol{x})\lambda}{\Delta t} \, d\ell \tag{SX-3}$$

Although $\widehat{\Lambda}_{\mathrm{AFAT}} \neq \Lambda$ for general $v(\boldsymbol{x})$, for many situations we expect it to remain a reasonable estimator.
