# Supplementary material for: Comparing Accuracies of Length-Type Geographic Atrophy Growth Rate Metrics Using Atrophy-Front Growth Modeling
Source: Ophthalmol Sci. 2022 Apr 14;2(3):100156. doi: 10.1016/j.xops.2022.100156 (PMC9560575; doi:10.1016/j.xops.2022.100156)
Supplement: Figure-S1 [file mmc11.pdf]

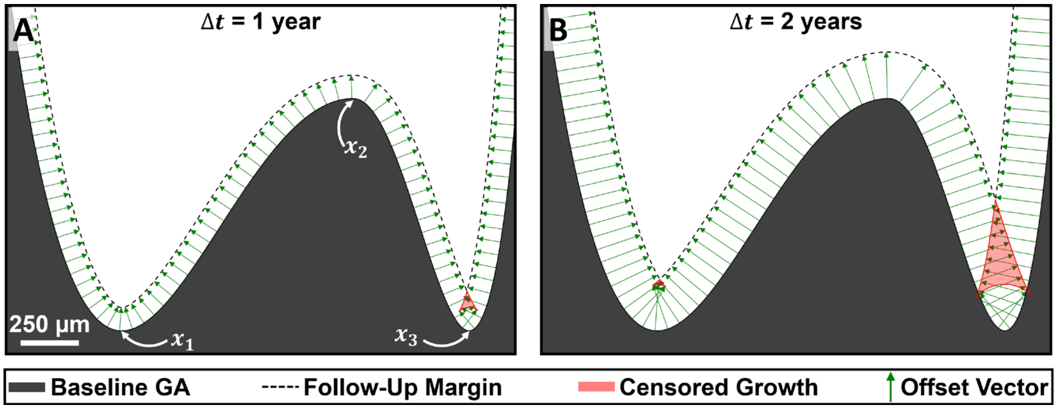

Figure S1. Illustration of the violation of Eq. SIII-1 due to intrafocus merging. The simulated baseline margins of both panels are undergoing isotropic growth at $v = 0.1$ mm/year. Three margin points, $\boldsymbol{x}_1, \boldsymbol{x}_2, \boldsymbol{x}_3 \in \partial G(t_b)$, are noted in panel A. Note that the offset vectors (green arrows) have lengths of $v\Delta t$. For concave margin segments (e.g., $\boldsymbol{x}_1$ and $\boldsymbol{x}_3$), depending on the magnitude of curvature and the offset distance $v\Delta t$, lesion growth may result in intrafocus merging (shaded red). In contrast, convex margin segments (e.g., $\boldsymbol{x}_2$) never undergo intrafocus merging.
