# Supplementary material for: Comparing Accuracies of Length-Type Geographic Atrophy Growth Rate Metrics Using Atrophy-Front Growth Modeling
Source: Ophthalmol Sci. 2022 Apr 14;2(3):100156. doi: 10.1016/j.xops.2022.100156 (PMC9560575; doi:10.1016/j.xops.2022.100156)
Supplement: Figure-S2 [file mmc12.pdf]

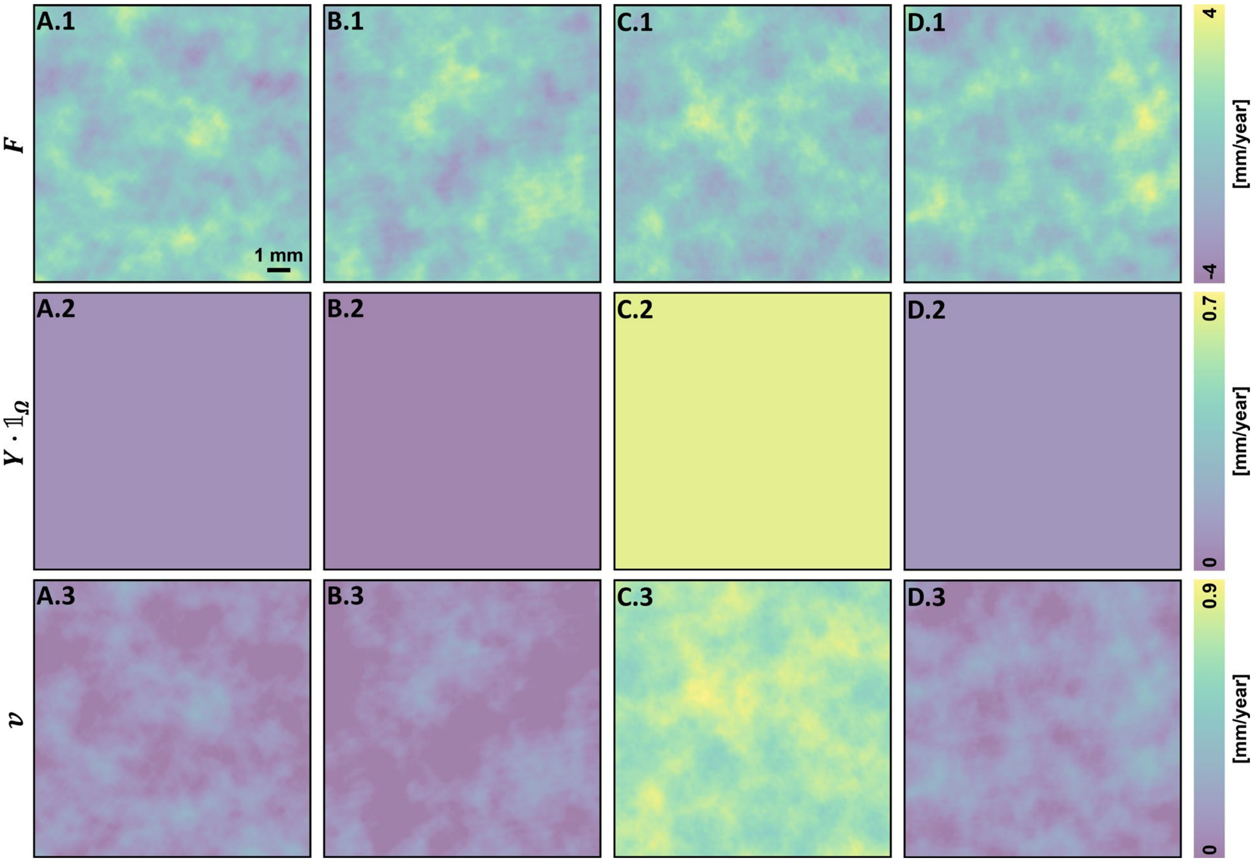

Figure S2. Illustration of random growth field generation. Columns A-D illustrate the construction of four different random fields, which were all used in this study. The first row corresponds to the Gaussian random field F (Eqs. SVI-1 and SVI-2). The second row corresponds to an isotropic growth field $Y \cdot \mathbb{1}_{\Omega}$, where the scale of Y (Eq. SVI-1) is exponentially distributed; $\mathbb{1}_{\Omega}$ is the unity function over the image domain Ω. The third row corresponds to v (Eq. SVI-1), generated with the scaling constants specified in the text. Note that the color bars have different scales for the three rows.
