# Supplementary material for: Comparing Accuracies of Length-Type Geographic Atrophy Growth Rate Metrics Using Atrophy-Front Growth Modeling
Source: Ophthalmol Sci. 2022 Apr 14;2(3):100156. doi: 10.1016/j.xops.2022.100156 (PMC9560575; doi:10.1016/j.xops.2022.100156)
Supplement: Figure-S3 [file mmc13.pdf]

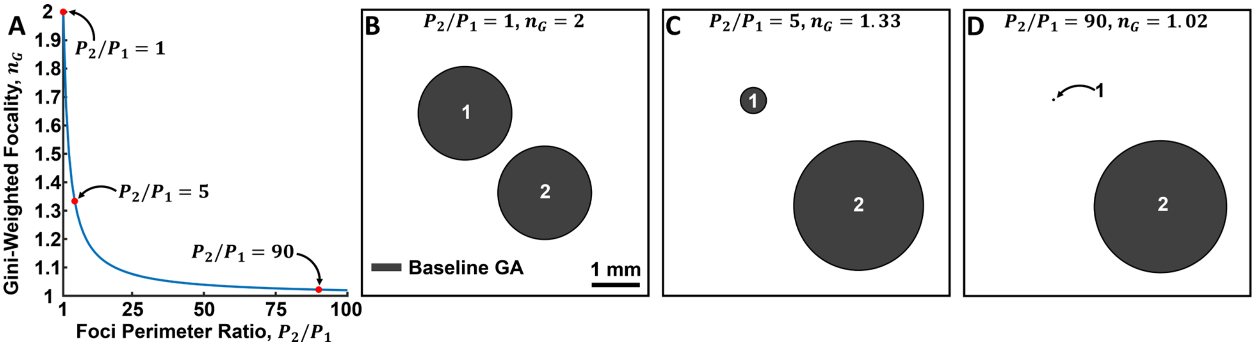

Figure S3. Illustration of Gini-weighted focality for different bifocal lesions. Specifically, we consider the situation of a 6 mm^2 lesion comprised of one focus with perimeter P_1 and a second focus with perimeter P_2. (A) Gini-weighted focality as a function of the ratio of the perimeters of the two foci. The three red circular markers correspond to three different perimeter ratios, which are visualized in panels B-D.
